# Trends in childhood leukemia incidence in urban countries and their relation to environmental factors, including space weather

**DOI:** 10.3389/fpubh.2024.1295643

**Published:** 2024-05-02

**Authors:** Olga Khabarova, Sergey K. Pinaev, Vladimir V. Chakov, Alexey Ya. Chizhov, Olga G. Pinaeva

**Affiliations:** ^1^Raymond and Beverly Sackler Faculty of Exact Sciences, Tel Aviv University, Tel Aviv, Israel; ^2^Far Eastern State Medical University, Khabarovsk, Russia; ^3^Far East Forestry Research Institute, Khabarovsk, Russia; ^4^Khabarovsk Federal Research Center, Far Eastern Branch of the Russian Academy of Sciences, Khabarovsk, Russia; ^5^Peoples' Friendship University of Russia, Moscow, Russia

**Keywords:** childhood leukemia, environment, gas pollutants, space weather, car pollution, fire pollutant, carcinogenesis

## Abstract

Leukemia is the most common cancer in children. Its incidence has been increasing worldwide since 1910th, suggesting the presence of common sources of the disease, most likely related to people’s lifestyle and environment. Understanding the relationship between childhood leukemia and environmental conditions is critical to preventing the disease. This discussion article examines established potentially-carcinogenic environmental factors, such as vehicle emissions and fires, alongside space weather-related parameters like cosmic rays and the geomagnetic field. To discern the primary contributor, we analyze trends and annual variations in leukemia incidence among 0-14-year-olds in the United States, Canada, Australia, and Russia from 1990 to 2018. Comparisons are drawn with the number of vehicles (representing gasoline emissions) and fire-affected land areas (indicative of fire-related pollutants), with novel data for Russia introduced for the first time. While childhood leukemia incidence is rising in all countries under study, the rate of increase in Russia is twice that of other nations, possibly due to a delayed surge in the country’s vehicle fleet compared to others. This trend in Russia may offer insights into past leukemia levels in the USA, Canada, and Australia. Our findings highlight vehicular emissions as the most substantial environmental hazard for children among the factors examined. We also advocate for the consideration of potential modulation of carcinogenic effects arising from variations in cosmic ray intensity, as well as the protective role of the geomagnetic field. To support the idea, we provide examples of potential space weather effects at both local and global scales. The additional analysis includes statistical data from 49 countries and underscores the significance of the magnetic field dip in the South Atlantic Anomaly in contributing to a peak in childhood leukemia incidence in Peru, Ecuador and Chile. We emphasize the importance of collectively assessing all potentially carcinogenic factors for the successful future predictions of childhood leukemia risk in each country.

## 1 Introduction

Leukemia is the most common form of malignant neoplasms in children. The world average standardized incidence rate of childhood leukemia is 46.4 cases per 10^6^ person-years (4.64 per 100,000), and it is increasing worldwide (1). Spatially, childhood leukemia presents notable irregularity across the globe [see data from International Agency for Research on Cancer https://iicc.iarc.fr/results/comparative-tables/ and (2)]. Fore example, the age-standardized incidence rate of leukemia was registered at the level of 4.54 cases per 100,000 in China in 2005–2017 with an increasing trend of 1.9% (3), and the corresponding data for Iraq collected for 2000–2019 show the rate of 3.45 with a growing trend of 1.23% per year in 2000–2009 (4). In 1993–2010, the rate was 3.96–4.32 per 100,000 in Japan and 4.58–4.80 per 100,000 in England (5). The age-standardized incidence rate of acute lymphoblastic leukemia, the most common type of childhood leukemia, was 5.33 per 100, 000 in Brasil in 1997–2004 (6), and 2.25 per 100,000 in Iran in 2006–2014 (7).

**Figure 1:**
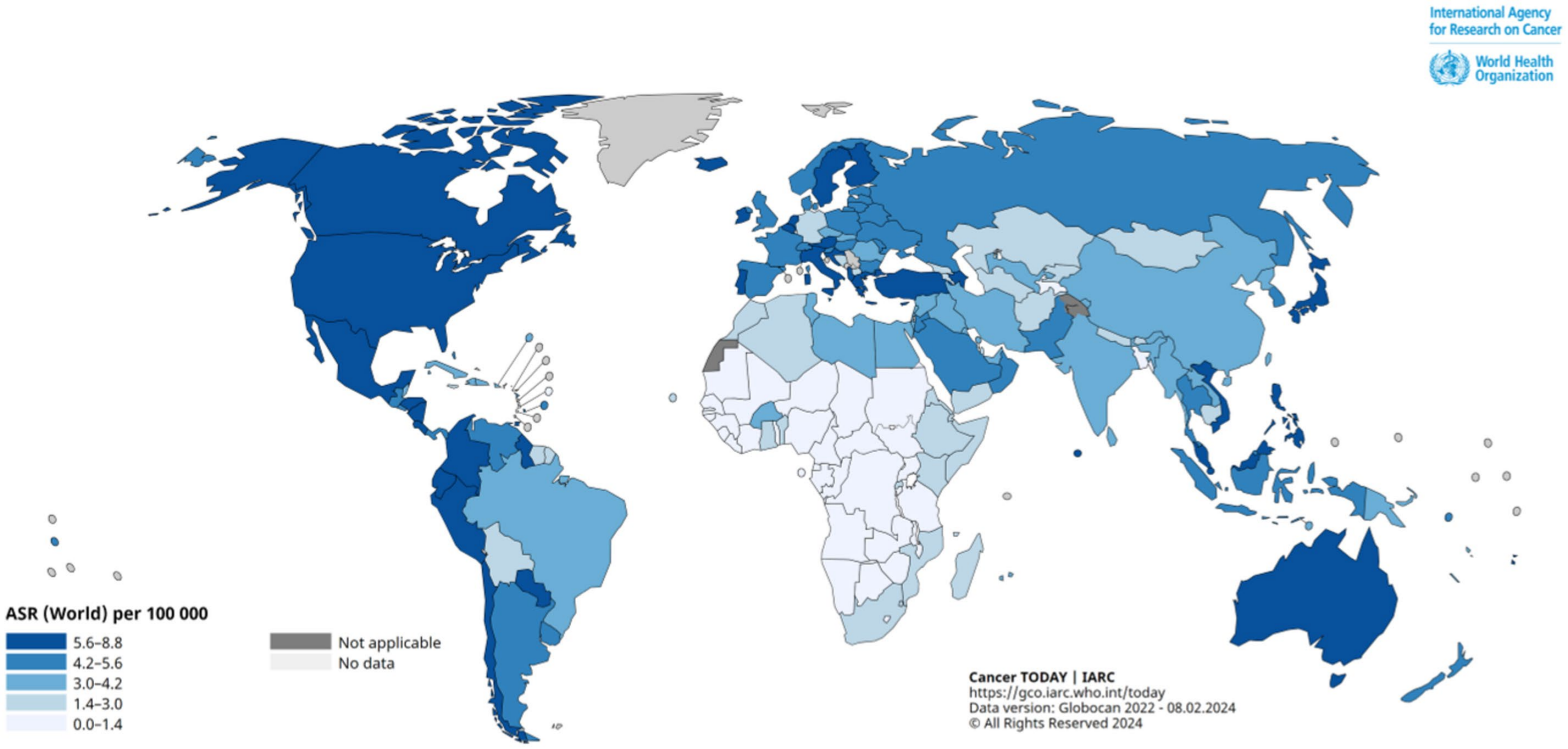
Global Age-Standardized Leukemia Incidence Rate (ASR) map, age 0–14, in 2022. Provided by the Global Cancer Observatory (GLOBOCAN) https://gco.iarc.fr/en.

In Figure 1 obtained from the Global Cancer Observatory (GLOBOCAN) https://gco.iarc.fr/en, the age standardized incidence rate (ASR) per 100,000 is shown with grades of blue. The darkest blue corresponds to the largest ASR values above 5.6 per 100,000. The GLOBOCAN or analogous resources collecting data from over the world, like Healthdata,^1^ are very useful but should be approached with care due to using limited databases from certain countries, which may lead to controversies, as one may find by comparisons with studies on leukemia statistics in individual countries. Meanwhile, the general GLOBOCAN picture of the leukemia incidence is close to reality. The map for year 2022 shown in Figure 1 illustrates that the global childhood leukemia distribution does not rigidly align with national borders. In high-income countries boasting robust medical infrastructure, leukemia is regarded as a manageable condition, and survival rates tend to closely align with the nation’s economic status (8), but incidence rates do not correspond to it or the quality of medical care. Despite extensive endeavors by the medical community, the underlying reasons for this irregular spatial distribution and the growing incidence rate persist elusive, and leukemia remains a very painful public problem.

It is important to acknowledge that our understanding of the global situation may be limited due to inadequate detection of leukemia cases and insufficient data collection in less developed countries or in countries with political regimes that prevent showing real statistics of the disease rate. This limitation may create a perception that countries rich in resources and with a well-established public healthcare system show a higher incidence rate of childhood leukemia, while those with fewer resources demonstrate lower rates of the disease (e.g., 6) Meanwhile, recent data from the International Agency for Research on Cancer https://iicc.iarc.fr/results/comparative-tables/ suggest that the ASR is highest in Peru (6.29 per 100,000), Ecuador (6.25 per 100,000) and Chile (6.21 per 100,000), among which only Chile is ranked by the World Bank as a high-income country. GLOBOCAN largely supports this assertion, highlighting South American countries as the most afflicted by childhood leukemia. Given the fluctuating list of top-affected countries from year to year and from source to source, it is reasonable to supplement the examination of comparisons of absolute values typical for different countries with an analysis of leukemia trends.

A rise in mortality rates attributed to leukemia among children has been documented in England and Wales, commencing from the years of the second industrial revolution, with initial data recorded in the 1910s (9). Given that mortality data from the early 20th century can serve as a proxy for incidence rates for the period when an adequate treatment was impossible, this trend suggests a commencement of a sustained rise in the number of children affected by leukemia. The study showed that children aged 0–4 years were statistically at higher risk, and mortality rates for acute lymphatic leukemia have a maximum at age 3 years in both sexes. The peak of mortality/incidence rate at ages 3–4 first reported in (9) was found later in different countries over the world (e.g., 3, 6, 10). One of the earliest databases on childhood leukemia in Europe shows a growth of the incidence rate in Denmark beginning from 1940th (11). By the early 1940s, the infantile peak of incidence appeared in US white boys, by the late 40s – in US white girls, in the early 1960s – in Japanese children, and to a lesser extent – in US African Americans (12, 13). In the 1950–60s, an increase in the incidence of childhood leukemia was also noted in Russia (12). In Hokkaido Prefecture, Japan, the frequency of acute lymphoid leukemia in children increased by 3 times from 1969 to 1993 (14). Studies of the incidence of childhood leukemia in southern Thailand and the United States (SEER 9) in the 1990–2010s showed a general upward trend, with higher levels of acute lymphoid leukemia in the US and acute myeloid leukemia in Thailand (15). Montero-Montoya et al. (16) have characterized the 21% increase in the incidence of leukemia in children in Mexico in 2000–2004 as an epidemic, compared to levels from 1980 to 1984.

Besides purely genetic origins of leukemia, the following factors are known to be associated with a high risk of childhood leukemia: radiation exposure, exposure to chemicals or chemotherapy drugs, parental smoking and alcohol consumption, blood disorders and immune system suppression (e.g., 17, 18). Recent review (19) claims that low doses of ionizing radiation in early childhood and general pesticide exposure during maternal preconception/pregnancy are strongly associated with childhood acute lymphoblastic leukemia, while other potential risk factors, such as electromagnetic field exposure (such as living near power lines), living near nuclear facilities, and various chemical exposures, show less evidence of association.

The main abovementioned factors contributing to leukemia vary from country to country and do not exhibit consistent trends. While statistically and experimentally proven, acknowledging these factors do not resolve the issue of the concurrent rise in leukemia incidence observed in multiple countries, as discussed earlier. In other words, while these factors undoubtedly contribute to leukemia, they may not fully account for the simultaneous variations and growth observed in leukemia incidence trends. According to the International Agency for Research on Cancer in children under 5, the incidence rate of acute lymphoid and acute myeloid leukemia continued to increase in many regions of the world in 1988–2012 (20). A continued growth in the incidence of all forms of malignant neoplasms in children, including leukemia, is predicted up to 2030, as it follows from the data analysis results for statistics gathered from 1971 to 2013 (21). Basic logic suggests that certain factors contributing to leukemia are likely shared among all countries experiencing economic growth and polluting the environment almost proportionally to it. Therefore, it is reasonable to study possible links of leukemia with purely environmental factors, which could play a role in shaping or influencing disease patterns.

Atmospheric pollution from smoke of any kind, including that from burning rubber, agricultural waste and tobacco smoke is known to be a significant contributor to the occurrence of childhood leukemia (see 19, 22–27 and references therein). A correlation has been found between the frequency of leukemia incidence in children and smoke from forest fires (26). It is believed that the children’s health damage caused by pollutants may begin from their parents being exposed several years before giving birth.

There is also evidence for a similar risk caused by benzene and gasoline exposure (16, 25). Chronic exposure to benzene associated with vehicle exhaust gasses, namely, polycyclic hydrocarbons and benzene, are known as major environmental risk factors for the occurrence of leukemia. A case–control study has shown that living near oil and gas production sites is associated with leukemia (24). There is a significant correlation between the distance from the place of residence to a gas station and the leukemia rate in children (25). Similar consequences are caused by benzene and gasoline exposure on future parents due to environmental pollution with aromatic hydrocarbons from oil refineries, paint and varnish enterprises and oil waste landfills (16, 24, 25).

Another potentially dangerous but poorly studied factor affecting young people and their parents is space weather. It is known that its extreme changes may cause different diseases and provoke acute exacerbation of existing health issues (28–32). An echo of solar storms is mainly dangerous because of bio-effective variations in the geomagnetic field at various temporal scales and increases in the intensity of secondary cosmic rays born in the terrestrial atmosphere (29, 33–36). Enhancements in the flux of energetic particles of the solar origin and galactic cosmic rays are especially strong in polar regions and may represent a serious problem for inhabitants of countries to where the auroral ovals reach. Since cosmic ray intensity increases with altitude, cabin crew members statistically face a larger risk of having cancer relative to the general population (37), and there also is a similar danger for the people spending a lot of time in the mountains. It has been found that there is a direct relationship between the frequency of childhood leukemia incidence and exposure to ultraviolet radiation (38), diagnostic X-rays and natural background ionizing radiation (39). The relationship between the solar activity reflected in the so-called Wolf numbers of sunspots and the incidence of leukemia in cohorts of young children aged 0–14 years has been claimed in several studies (26, 40). There is evidence for a link between all potentially adverse factors mentioned above and cancer, including leukemia, although more studies are necessary to understand the mechanisms behind it (39).

It is important to consider the environment in which children and their parents live in a wide sense, taking into account several factors associated with the childhood leukemia risk. The childhood leukemia incidence rate is increasing worldwide. Further studies of possible causes of this disease are important for developing effective measures for its prevention. The aim of this work is to analyze general trends and annual fluctuations in the leukemia incidence rate in children in the USA, Canada, Russia, and Australia and find possible relationships between them and known potentially carcinogenic environmental factors. According to the World Bank data at: https://data.worldbank.org/indicator/SP.URB.TOTL.IN.ZS, all these countries have the index of urbanization over 70% (the USA –83.1%, Australia –86.5%, Canada –81.8%, and Russia –75.1%). A comparative analysis of long-term leukemia trends linked to the data for transport ownership and fires in the four countries mentioned above has not been previously carried out for a long period (1990–2018) considered in this work.

This study introduces novel data for Russia. Over the past decade, experts have extensively examined the trends and potential origins of childhood leukemia in the USA, Canada, and Australia. They have highlighted leukemia as a significant concern due to its increasing incidence, in contrast to some other types of cancer that have either remained stable or declined [see (40–42) and https://www.aihw.gov.au/reports/children-youth/australias-children/contents/health/cancer-incidence-survival]. Despite numerous publications addressing this issue in the USA, Canada, and Australia, this topic remains inadequately explored in Russia. No prior comparative analyses or trend assessments have been conducted for Russia, hindering a comprehensive understanding of the country’s position regarding this global health concern. Systematic statistical data on childhood leukemia at the national level in Russia is lacking, although there exist noteworthy international-level publications on childhood leukemia in the country (43–45). Russia is known for its slower economic progress compared to several other urban nations. This distinctiveness suggests the likelihood of observing trends akin to those identified years ago in the largest urban countries. Results of this study provide a unique opportunity to examine hypotheses concerning the origins of leukemia in historical contexts across the USA, Canada, and Australia.

Being primarily a discussion article, this manuscript focuses on a comparative analysis of childhood leukemia incidence trends and delves into potential causes of the disease. It includes speculations on less-explored factors like space weather-related effects, often overlooked and requiring attention from various scientific communities. We investigate the hypothesis concerning the carcinogenic effects of not only exceptionally intense radiation exposure but also regular exposure to cosmic rays across various global locations. A possible evidence for linked leukemia and cosmic ray intensity trends is provided for the locations in Russia and the USA where local cosmic ray intensity measurements are carried out for many years. In support of this concept, we present arguments for potential carcinogenic effects of cosmic rays and the protective influence of the geomagnetic field. This is demonstrated by comparing general childhood leukemia statistics with the geomagnetic field intensity for additional 45 countries besides the USA, Canada, Russia, and Australia. We also show that the peak in the disease occurrence observed in South America may be linked to the South Atlantic magnetic field anomaly.

## 2 Materials and methods

Both the linear and polynomial regression methods are used to illustrate rising trends in the childhood leukemia in different countries. The methods follow the principle: *y* = A_0_ + A_1_*x*^1^ + A_2_*x*^2^ + … + A_n_*x*^n^, where *y* represents the dependent variable, *x* is the independent variable, a_0…_a_n_ are the coefficients, and *n* denotes the polynomial order. In the case of a linear regression, *n* = 1. Details of the fitting results, including the coefficient of determination, the confidence interval and residual values, are given in figures and tables of the Supplementary material S1, and the manuscript figures show key information such as the formula and the slope. Although the linear approximation gives a general impression about the direction of the trend, the behavior of the curves shows some non-linear features. In Supplementary material S1, we show results of non-linear fitting of the disease rate curves to a higher-degree polynomial, for which polynomial regression constructing a polynomial equation to approximate the relationship between variables in a dataset can be employed. The Levenberg–Marquardt algorithm is used for curve-fitting, i.e., for accurate fitting a nonlinear model to observed data, as relationships between data points are nonlinear in all the cases discussed in the manuscript. The algorithm adjusts model parameters iteratively to minimize the difference between observed data and model predictions by combining gradient descent and Newton’s methods. The corresponding references for the Levenberg–Marquardt algorithm and Newton’s methods are given in the Supplementary material S1. Additionally to the trend analysis, we calculate the average annual growth rate in percentage as (R_year + 1_ – R_year_)⋅100/R_year_, where “year” is some year and “year+1” is the next year, in order to show variations in the parameter through the period considered in this study.

The information gathered at the population level is very important since it pictures a country profile for the corresponding phenomenon. When the length of the rows for yearly averaged data is not large (1990–2018 in our case), the comparative study is only able to show results of a basic correlation analysis that allows checking hypotheses about potential harmful external agents causing leukemia in children. In order to understand which environmental factors can cause stable growing trends in childhood leukemia, we have compared the data series for the leukemia incidence rate in the four countries with data for fires and analyzed statistics for car ownership. Checking the idea about a possible space weather impact, we also analyze data for the parameters reflecting the solar and geomagnetic activity, namely, the sunspot number, the Kp index geomagnetic activity, the geomagnetic field and the cosmic ray intensity, a description of which is given below.

Correlation analysis has been selected as the primary analytical approach for assessing relationships between the leukemia incidence rate and environmental parameters in this study. The following considerations led us to making this choice. Given the limited sample size (far below 40 points), traditional hypothesis testing methods may lack adequate statistical power to detect meaningful associations between variables. Correlation analysis offers a more robust alternative, focusing on the strength and direction of relationships without strict dependence on sample size. Hypothesis testing often relies on the assumption of normality, which may be challenging to meet with small sample sizes. Correlation analysis is less sensitive to distributional assumptions, making it suitable for exploratory analyses where data normality cannot be assumed. In situations where the objective is to explore potential associations between variables or generate hypotheses for further investigation, the correlation analysis provides a flexible and informative approach. It allows for the identification of patterns or trends in the data without requiring pre-specified hypotheses. Correlation coefficients offer a straightforward measure of the strength and direction of relationships between variables, facilitating clear and concise interpretation of results compared to traditional hypothesis tests. Despite small sample sizes, correlation analysis remains robust, particularly when examining strong relationships between variables. It enables researchers to glean valuable insights and identify potential avenues for further research or decision-making, even in the absence of statistical significance. Therefore, when dealing with a small sample size, correlation analysis offers a flexible and informative approach to understanding relationships between variables, making it a valuable tool in data exploration and hypothesis generation.

Data on the incidence of all types of leukemia in children aged 0–14 in the US, Canada, and Australia and the necessary information on methodology can be reached by direct links provided in the Appendix. Data for the USA are taken from the Cancer Statistics Explorer Network of the National Cancer Institute and for Canada – from the National Cancer Institute and Statistics Canada. Data for Australia are provided by the Cancer Council Queensland and taken from the Australian Childhood Cancer Registry funded and managed by Cancer Council Queensland. Incidence rate of Childhood Leukemia in New Jersey is taken from New Jersey’s Public Health Data Resource of New Jersey Department of Health.

Note that the annual number of children diagnosed with leukemia is given for the US and Australia for the entire cohort of children aged 0–14 and for all types of leukemia, while the corresponding data for Canada are given for children of 0–4, 5–9, and 10–14 years old and systematized by a leukemia type. Therefore, for Canada, one needs to gather and summarize data for incidences of the acute myeloid leukemia, acute lymphocytic leukemia, chronic myeloid leukemia, chronic lymphocytic leukemia, and other leukemia types for all children aged 0–14 from the corresponding website.

Unique data on the leukemia incidence rate in Russia are presented in Supplementary Table S1, along with the description of the method of making the corresponding diagnosis. The leukemia incidence rate data in children aged 0–14 years in Russia in 1997–2018 are obtained from the public annual official reports of the P. Hertsen Moscow Oncology Research Institute.^2^ Childhood leukemia data in Moscow (1990–2018) and Russia (1990–1996) are gathered from the Information and Analytical Database System on Oncology based on the “Software IAS database of federal statistical reporting on oncology” developed by P. Hertsen Moscow Oncology Research Institute (non-open-access). The diagnosis of acute lymphoid leukemia in Russia is made out according to the standards of medical care in the Russian Federation posted on the official website of the National Society of Pediatric Hematologists and Oncologists.^3^

All data on leukemia incidence are used in a form of rough rates for both sexes and all races per 100,000 population per year, i.e., the number of new cases is divided by children population and multiplied by 100,000 (see Supplementary Table S1). Global population census data for all the countries are from Microtrends and United Nations – World Population Prospects. The historical number of children between the ages 0 to 14 for the USA, Canada and Australia can be found in the United Nations Population Division’s World Population Prospects (Worldbank). Children population census data for Russia listed in the Supplementary material S1 are provided on Russian Federal State Statistics Service (Rosstat).

Fires can be dangerous as they produce a lot of carcinogenic gasses. Data on the land areas affected by fires in the US are provided by the National Interagency Fire Center, data for Canada are from the National Forestry Database, data for Australia are from Canadell et al. (46), and the analogous data for Russia are from the Federal Forestry Agency (in Russian). The information is available in Supplementary Table S2.

To examine a possible carcinogenic impact of cars as the most common pollutants, statistics of car ownership have been taken into account. We use data for the total number of vehicles per 1,000 people, available by links provided in Appendix. The number of cars owned in the USA is from the Federal Highway Administration of the US Department of Transportation, and the same information for Russia is from the official Statistical governmental office, so-called Rosstat (in Russian). The statistics exclude two-wheel-vehicles. Analogous data for Canada is unavailable in a pre-processed form; therefore we calculated the car number parameter using detailed statistics for different categories of the vehicles provided by Statistics Canada. The corresponding parameter was computed as the number of cars normalized to the number of people in population and multiplied by 1,000. Motor Vehicle Census data for Australia are collected from annual reports of the Australian Bureau of Statistics. The number of four-wheel vehicles registered annually in the four countries can be found in Supplementary Table S3. Note that no data for Canada is available before 1999 on the official resources.

An analysis of the relationship between the leukemia incidence rate and the annual average number of sunspots (so-called Wolf numbers) and geomagnetic activity Kp index reflecting space weather changes has also been carried out. Sunspots are linked to active regions ejecting geoeffective streams, and their number reflects the activity of the Sun and follows the 11-year solar cycle. In turn, the Kp index is a way to measure disturbances in the terrestrial magnetic field caused by solar activity. This is one of the most popular indicators used to understand if the variable geomagnetic field is calm or disturbed. The index characterizes the maximum disturbance of the horizontal component of the Earth’s magnetic field in a specific three-hour period measured at various geomagnetic observatories over the globe. It ranges from 0 to 9, with 0 indicating very little geomagnetic activity and 9 signifying an extremely strong geomagnetic storm. The behavior of the sunspot numbers is smooth in comparison with highly variable Kp index. Data on the solar and geomagnetic activity are obtained from the official NASA OMNI database.

To analyze a possible link between the childhood leukemia rate at the global level and space weather factors, we use the ASR data from the International Agency for Research on Cancer https://iicc.iarc.fr/results/comparative-tables/ compared with the intensity of the total magnetic field of the Earth calculated for each country using the NOAA World Magnetic Model (WMM) https://www.ngdc.noaa.gov/geomag/calculators/magcalc.shtml?useFullSite=true#igrfwmm as presented in Supplementary Table S4.

Data from neutron monitors reflect variations in the intensity of high-energy cosmic rays (energetic particles) bombarding the Earth. Such particles represent a potential harm to living objects, affecting those at the cell level. Cosmic rays of galactic and solar origin interact with the terrestrial atmosphere and produce a so-called atmospheric cascade of secondary energetic particles that can be measured by ground-based neutral monitors (see https://www.nmdb.eu/public_outreach/en/).

Neutron monitors are ground detectors sensitive to cosmic rays penetrating the Earth’s atmosphere, typically with energies ranging from 0.5 to 20 GeV. Galactic cosmic rays (GCRs), originating from remnants of stellar explosions and active galaxies, travel from the outer heliosphere towards the Sun with minimum speeds of just two thirds of the speed of light (29). These GCRs create a backdrop of high-energy protons and helium nuclei that neutron monitors often measure. During periods of high solar activity (characterized by the high sunspot numbers), the Sun emits an increased volume of accelerated particles into the heliosphere. Accelerated solar energetic particles (SEPs) have energies up to the MeV range, while the most energetic SEPs, with energies surpassing the MeV range, are referred to as solar cosmic rays (SCRs). After strong solar flares, neutron monitors detect heightened fluxes of SCRs, known as “ground level enhancements” (GLEs). The intensities of GCRs and SCRs anti-correlate over the solar cycle. Cosmic ray intensity within the Earth’s atmosphere exhibits variations linked to both altitude and latitude. The flux of cosmic rays is notably influenced by altitude, increasing with higher locations due to reduced atmospheric shielding. All calculators of the radiation dose take the elevation above the sea level into account (e.g., https://www.epa.gov/radiation/calculate-your-radiation-dose). Latitude also significantly affects the intensity of cosmic rays reaching the ground because the magnetic field of the Earth impacts trajectories of cosmic rays. Proximity to the polar regions is associated with the stronger influence of cosmic rays on biological objects due to the terrestrial magnetic field configuration, i.e., northern countries are more affected by high-energy particles than those located at low geomagnetic latitudes. In sum, it means that cosmic ray effects on humans are rather local, and, although the CR flux modulation by the 11-year solar cycle is obvious at all cosmic ray stations, for smaller temporal scale comparisons it is preferable to measure it near the location where a particular medical experiment is carried out. To make a preliminary analysis of a possible impact of cosmic rays on children, the Moscow (Russia) and Newark (the USA) neutral monitor station data are used in our study. The stations participate in the European Union’s FP7 programme, and the corresponding data can be found in the global Neutron Monitor Database (NMDB), as indicated in Appendix. The number of particle counts per second uncorrected for the atmospheric pressure is averaged per year. More information about the data can be found on the official NMDB website. The corresponding links to all databases mentioned above can be found in the Appendix.

## 3 Results

### 3.1 Childhood leukemia incidence trends in Australia, Canada, the USA, and Russia

**Figure 2:**
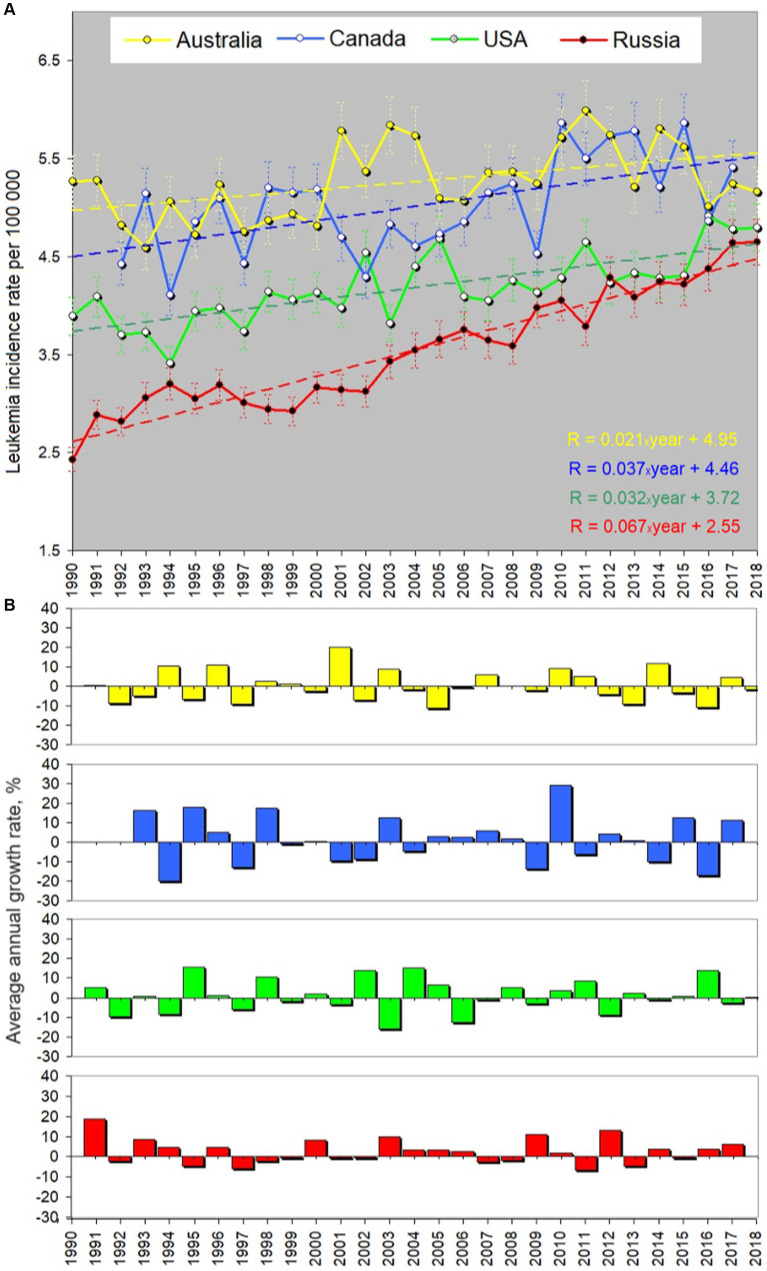
Yearly averaged leukemia incidence rate in children aged 0–14 (per 100.000 children) in four urban countries. All sexes, races and types of leukemia are considered. **(A)** Rates, errors and linear approximation. Yellow curve shows the course of the incidence rate in Australia, blue – in Canada, green – in the USA, and red – in Russia. Dashed lines of the corresponding colors reflect the linear trend line. **(B)** Average annual percentage growth rate. Colors correspond to **(A)**. “Year” in the equation in panels **(A)** is defined as 0 for 1990, 1 for 1991, etc. The coefficient before “year” in the trend equation characterizes the growth of the approximation line. Russia shows the fastest growth in the incidence rate (the increase is 67% for 30 years). Descriptive statistics and the approximation details can be found in the Supplementary material S1.

Figure 2A shows a comparison of the childhood leukemia incidence rate (R) trends for Australia, Canada, the USA, and Russia, and Figure 2B illustrates changes in the average annual growth rate in percentage. In Figure 2, yellow is for Australia, blue is for Canada, green is for the USA, and red is for Russia. Figure 2A shows the rate *R* and the corresponding linear approximation results displayed behind each curve in the form of thin dashed lines of the same colors. Basic descriptive statistics reflecting the general behavior of the incidence rate in each country and details about the linear approximation results are provided in a form of Supplementary Table S5 and Supplementary Figures S1–S4. It should be noted that although the linear approximation gives a general impression about a rising direction of the trend, higher-polynomial approximation may fit the curves better. As shown in the Supplementary material S1, the coefficient of determination varies from 0.2 to 0.6 with an exception for Russia (0.92). Fifth-degree polynomials offer a more satisfactory fit. As delineated in Supplementary Figures S5–S8, the coefficient of determination shows the best values for the fifth-degree polynomial (0.5–0.96), although a third-degree polynomial can be used too in order, for example, to involve this dependence in building a model in the future. Meanwhile, for a practical aim of comparison of trends, the linear approximation shown in Figure 2A is satisfactorily to be used since it reveals the clear general upward trend across all incidence rate curves. The height of each curve reflects the data collection specificity in respective countries and holds less significance compared to the curve’s steepness depicted by the approximation equation provided in the lower right corner of Figure 2A. In the equations, 1990 corresponds to year number 0, 1991 – to 1, etc.

The associated multiplier before “year” dictating the curve’s growth is distinct for each country. Its meaning is the slope of the approximation line or the first derivative. The corresponding values of the slope are given in Table 1. According to the approximation equations, Russia demonstrates a multiplier value of 0.067, contrasting with 0.021 for Australia, 0.037 for Canada, and 0.032 for the US. Notably, the USA and Canada exhibit similar trends (the blue and green dashed lines being nearly parallel), while the incidence of childhood leukemia in Russia increases at a rate twice as fast as that in the USA and Canada and three times faster than in Australia. This is an important finding, and we will discuss below why the number of children suffering of leukemia in Russia grows this way in comparison with the other countries.

**Table 1:** Analysis of trends and variations.

Country	1. Slope, incidence rate	2. Slope, area burnt	3. Slope, cars	4. Correlation: area burnt vs incidence rate	5. Correlation: cars vs incidence rate
US	0.0321	0.0798	0.0264	0.569	0.669
Russia	0.0668	0.0668	0.0939	0.638	0.961
Australia	0.0208	0.0869	0.0760	0.362	0.429
Canada	0.0366	0.0160	0.1097	0.115	0.611

**Figure 3:**
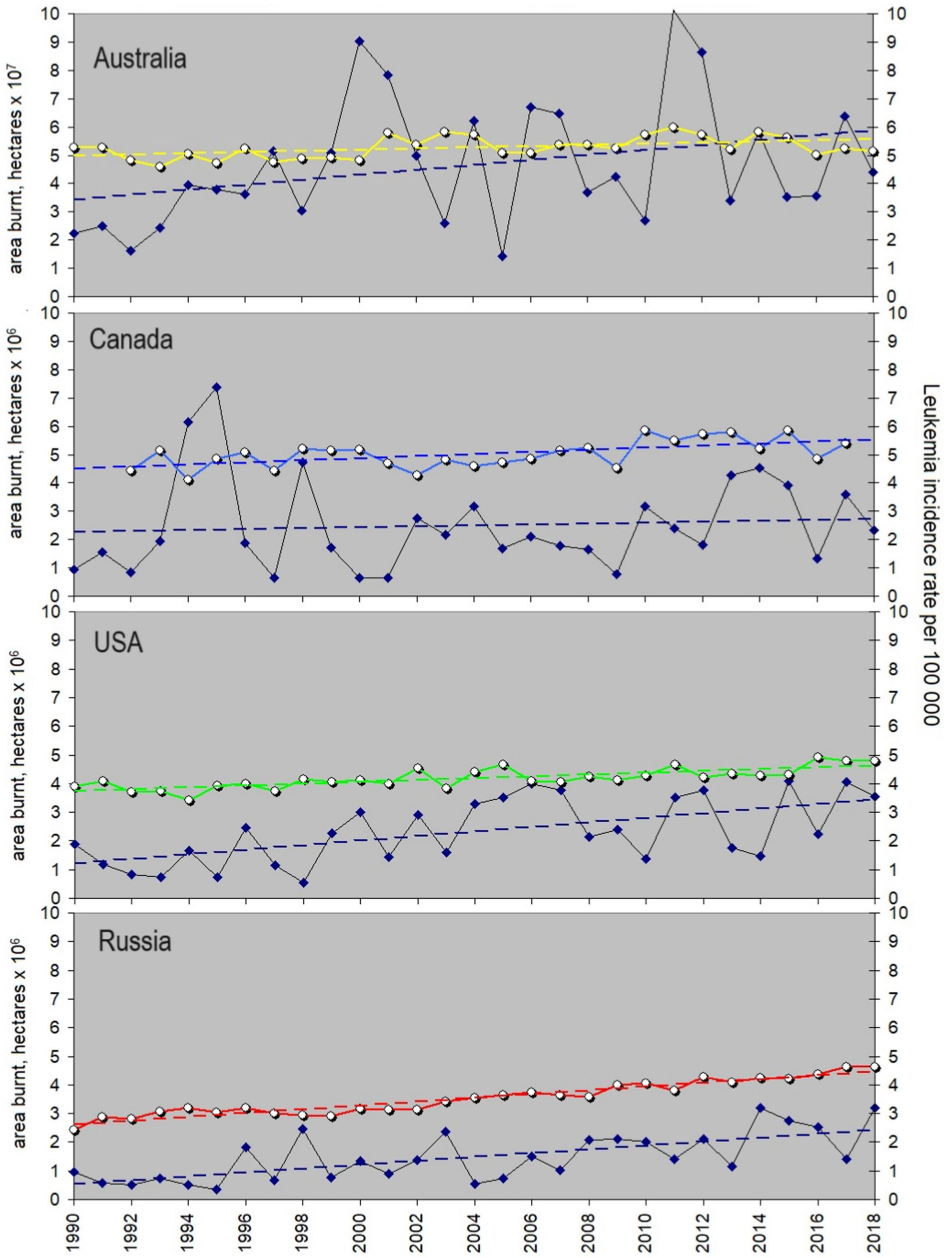
Yearly averaged leukemia incidence rate in children and its possible association with toxic gasses from fires. Areas burnt are a proxy of the volume of cancer-causing gasses impacting children and their future parents. From top to bottom: leukemia incidence rates in Australia, Canada, US, and Russia shown with colors analogous to Figure 2 vs. the areas burnt annually in each country (dark blue diamonds, black line). Linear trends are shown with dashed lines of the corresponding colors. Details of the trend slopes and correlation coefficients are given in Table 1, and the data are provided in Supplementary Table S2.

Slope (or the first derivative) is calculated as the difference between the last value of the linear approximation trend line and its first value divided by the total number of years during which the study is performed. The first, second and the third columns show the slope of the approximation lines of (1) the childhood leukemia incidence rate (see Figure 2), (2) the area devastated by fires (see Figure 3), (3) the relative number of vehicles registered (see Figure 5) in the corresponding countries. Correlation coefficients for comparisons of variations in the childhood leukemia rate vs. the areas burnt in fires are shown in column (4), and for the childhood leukemia rate vs. the relative number of vehicles – in column (5).

### 3.2 Possible carcinogenic impact of gasses from fires and cars

Figure 3 shows a comparison of the area devastated by fire (as a proxy of the fire-associated carcinogenic gasses) with the leukemia incidence rate for all the countries listed above. The latter is marked by the same colors as in Figure 2. One can find that variations in the area burnt are highly irregular. This is a well-known peculiarity determined by both natural processes like lightning strikes and human activity. Table 1 provides the respective slopes for each approximation line alongside the linear correlation coefficients between the area burnt and leukemia incidence rate, computed for each country. One can find that the area devastated by fires grows faster in Australia than in the other countries with the slope of 0.087 vs. 0.079 for the USA, 0.067 for Russia and merely 0.016 for Canada. The most important feature is that the slopes of the long-term trend of the leukemia incidence and the area burnt in Russia coincide precisely. The correlation coefficient between the leukemia rate and wildfire area curves is 0.64, which is the largest one among the corresponding pairs of curves. This may indicate a strong impact of gasses emitted by fires on children’s health in Russia.

**Figure 4:**
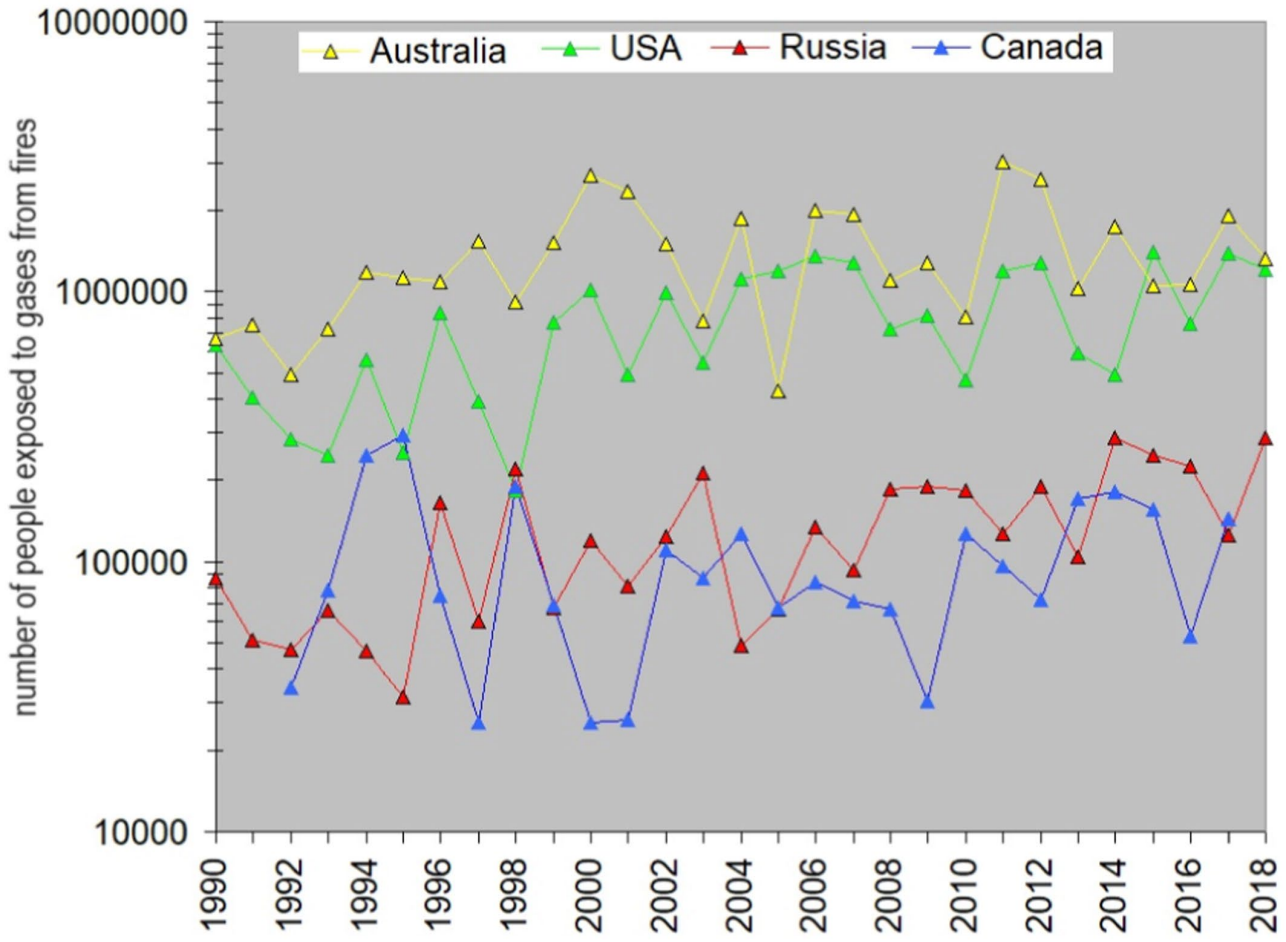
Estimated number of people exposed to pollutants emitted by wildfires in Australia, the USA, Russia, and Canada shown in Figure 3. Yellow is for Australia, green – for the USA, red- for Russia, and blue – for Canada.

Meanwhile, the leukemia incidence rate in Canada shows no possible tie with fires. The latter could indicate that the threshold for carcinogenic fire-related gasses, crucial for childhood leukemia development, is likely not being exceeded in Canada. This is potentially due to the country’s relatively stable fire situation seen from the slope value coupled with its low population density. Indeed, if we calculate the number of people exposed to gasses from fires in each country, it is easy to find that the numbers for both Russia and Canada are far below those typical for Australia and the USA (Figure 4). The number of people exposed can be estimated as the Area burnt (in km^2^) multiplied by the population density (people per km^2^). The rate is rather stable, so for the simplicity of estimations we take its values reached in 2010. According to https://www.worldometers.info/world-population, it was largest in the USA (34 people/km^2^) and smallest in Australia (3 people/km^2^). Despite Australia’s lower population density, the extensive wildfire-prone area poses a significantly higher risk to people compared to Canada (with a population density of 4 people/km^2^) or Russia (at 9 people/km^2^), and the curve in Figure 4 for Australia overlaps with that for the USA. One may expect that the correlation of the leukemia incidence rate with areas burnt is larger for the countries where people are exposed the most. However, the correlation coefficient for Russia does not follow this sequence. It means that other factors linked with fires may exist and affect the children in this country.

The trends observed suggest that exposure of children by fire gasses and occurrence of leukemia may be related, and if so, one may suppose that there is a long-term impact of fires on children and/or their future parents or a delay in the development of leukemia in children. It can be revealed by shifting the area-burnt curve back with respect to the leukemia curve and calculating the corresponding correlation coefficient. The cross-correlation test shows that a shift of the rows with respect to each other does not improve the situation much in the case of Canada, and r never exceeds 0.3 here. In contrast, a one-year shift reveals an *r*-value of 0.63 for USA data, a two-year shift boosts the correlation to 0.71 for Russia, and a three-year shift yields a correlation of 0.55 for Australia. Note that a time delay between the exposure and a start of the disease may vary, making it difficult to trace a direct link between the events. These results support an idea about fires as potentially cancer-inducing factors, especially taking into account a relatively high correlation between the analyzed data series in the USA and Russia. However, if such a connection exists, a direct impact of fires is hardly the only cause of childhood leukemia.

**Figure 5:**
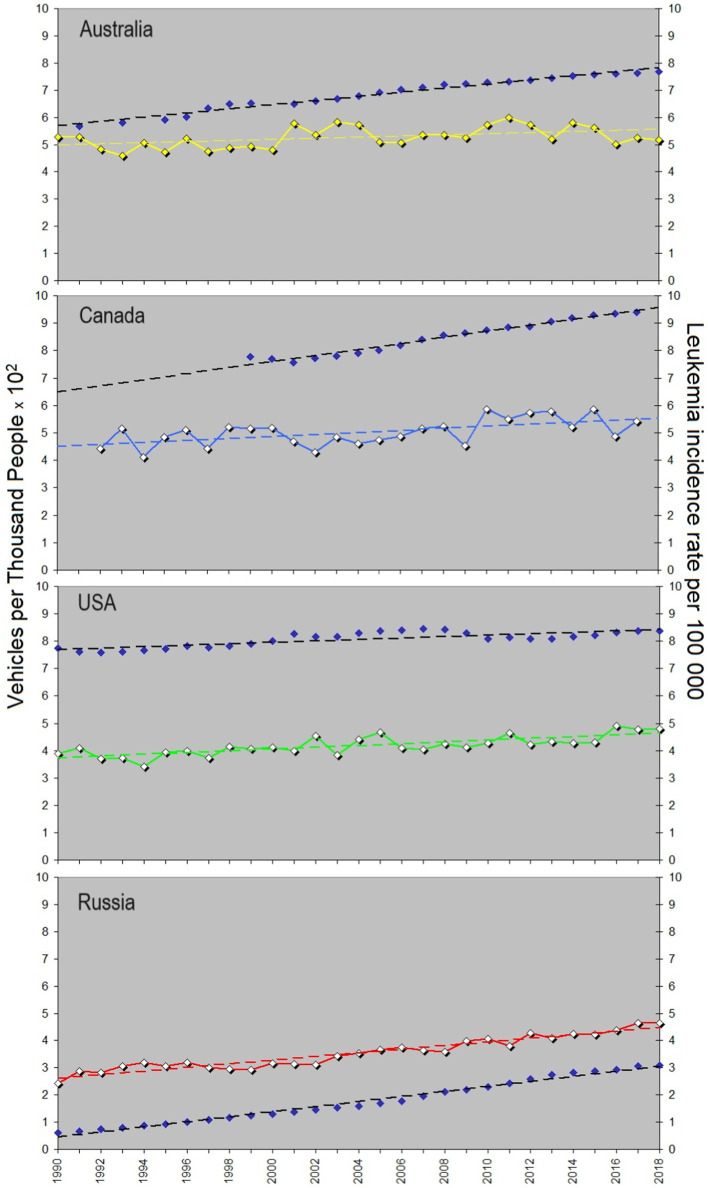
Yearly averaged leukemia incidence rate in children and its possible association with carcinogenic gasses from cars. Yellow curve shows the course of the childhood leukemia incidence rate in Australia, blue – in Canada, green – in the USA, and red – in Russia. Dark blue dots characterize the number of vehicles owned in the corresponding countries. Linear trends are shown with dashed lines, analogous to Figure 3. Details of the trend slopes and correlation coefficients can be found in Table 1, and the data are given in Supplementary Table S3.

Looking for trends similar to that observed for childhood leukemia, we present below data on the number of vehicles registered annually in the four countries, as shown in Figure 5, which is plotted analogous to Figure 3. Note that the original data are in the number of vehicles per 1,000 (see Supplementary Table S3), therefore the vehicle number per thousand people multiplied by 10^2^ shown in Figure 5 corresponds to the number of vehicles per 100,000 people, to be adequately compared with the leukemia rate calculated per 100,000. The trend’s slopes and correlation coefficients can be found in Table 1. As one can see, Canada’s car park grows faster than in the other countries, and the USA curve for vehicles registered shows the slowest growth. The correlation coefficient between the leukemia incidence and the car number varies from 0.43 (Australia) to 0.961 (Russia), which makes Russian population unprecedentedly sensitive to temporal variations in car-emitted gasses.

This suggests that emissions from cars significantly contribute to causing leukemia in children and directly impact them. The increase in the number of vehicles in urban areas poses a major challenge from an ecological standpoint. The cross-correlation analysis indicates a lagged response to variations in the number of cars in Australia, with the correlation coefficient increasing to 0.54 after a 2-year shift. No notable changes are observed when similar shifts are applied to other countries’ data. Comparing information above, one may conclude that gasses both from fires and cars may be significant contributors in the development of leukemia in children with a one-two year delay, with a larger impact of fumes from vehicles.

### 3.3 Possible impact of space weather, including cosmic rays and geomagnetic field

#### 3.3.1 General information about solar-terrestrial couplings and bio-effects of space weather

**Figure 6:**
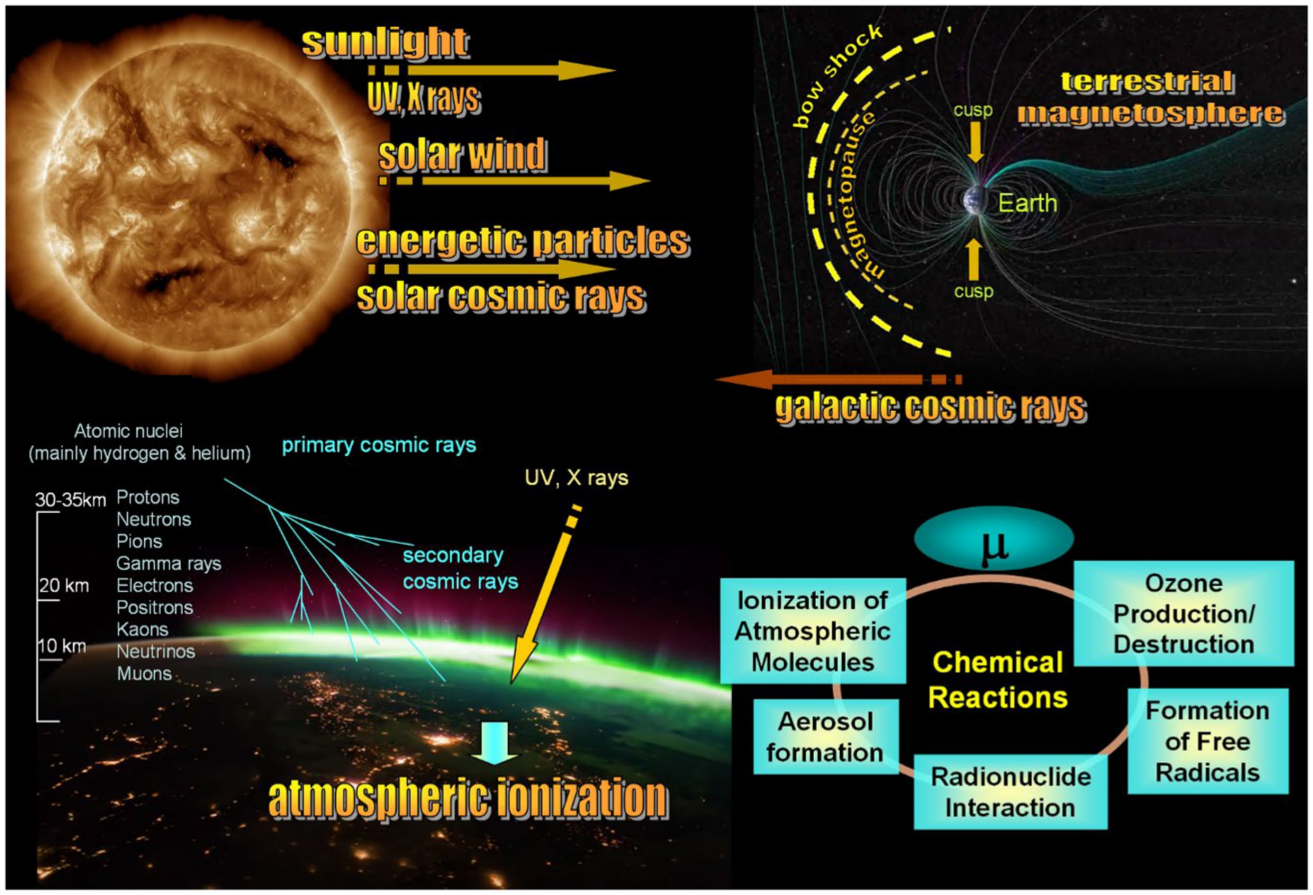
Key aspects of solar-terrestrial couplings and impact of cosmic rays on the atmosphere and organisms living on the Earth. Upper panel illustrates the primary factors connecting the Sun and Earth through the solar wind plasma, the terrestrial magnetosphere, and ionosphere. Two bottom sketches depict the physical and chemical processes that arise from space weather effects in the terrestrial ionosphere and atmosphere, potentially posing a carcinogenic risk. Sun’s impact on living organisms is always indirect, and finding an acting agent responsible for a particular bio-effect is the most difficult task of heliobiology.

Space weather is known to affect bio-objects in different ways (29, 30, 32, 47), but the main point about solar-terrestrial couplings is that nothing coming from the Sun impacts the ground and the terrestrial biosphere directly. The upper sketch in Figure 6 illustrates the main acting agents that Sun emits.

- Solar radiation spans the entire electromagnetic spectrum, defined by the “solar constant” at about 1.361 kilowatts per square meter on average, with minor fluctuations of around 0.2% during the solar cycle. Throughout history, human eyes have adapted to perceive sunlight, which aligns with the peak of the solar spectrum. However, the spectrum extends across all frequencies and energy ranges, encompassing ultraviolet (UV) and X-rays (Röntgen radiation).
- There is no complete vacuum between the Earth and the Sun. Instead, there exists the solar wind plasma, an extension of the expanding solar corona. This plasma is quasi-neutral and primarily comprises protons and electrons. A crucial attribute of the solar wind is its capacity to transport the interplanetary magnetic field, determining the effectiveness of the interaction between high-speed solar wind streams and the Earth’s magnetosphere causing geomagnetic storms.
- Energetic particles with energies above 100 MeV emitted during solar flares are called cosmic rays (SCRs) to differentiate them from less energetic particles, SEPs, of keV-MeV energies. The latter can be accelerated both in solar flares and at shocks driven by high-speed streams, coronal mass ejections. The other source of accelerated particles is shocks associated with stream interaction regions representing borders of high-speed flows from coronal holes. There are also mechanisms accelerating particles to MeV energies locally in the solar wind by magnetic reconnection (48).
- Cosmic rays of the galactic origin (GCRs) are high-energy particles originating from outside the solar system, likely from sources across the Milky Way and beyond. They stream from the edges of the heliosphere towards the Sun. Unlike solar cosmic rays, which intensity follows the solar activity cycle and peak at solar activity maximum, GCRs are swept away by the increased interplanetary magnetic field during solar maximum, therefore their flux anti-correlates with the solar cycle. SCRs are predominantly composed of protons and helium, and the GCR composition includes a diverse range of high-energy particles, such as protons, electrons, heavier nuclei, and even antimatter particles.

None of (i)–(iii) hits the ground directly. The terrestrial magnetosphere bounded by the bow shock, the downstream turbulent sheath and the magnetopause separating the sheath from the internal geomagnetic field, as shown in the upper sketch in Figure 6, deflects the solar wind and most energetic particles and cosmic rays. The only regions truly dangerous for biosphere are cusps, projected to the auroral ovals closer to the atmosphere, the places to where CRs and energetic particles can penetrate. The cusps practically represent holes in the magnetosphere in which lines of the geomagnetic field are directed towards the Earth surface, and the geomagnetic field cannot prevent propagation of dangerous particles coming from the Sun or accelerated in the solar wind. The rest agents associated with severe space weather are shielded by both the magnetic field deflecting charged accelerate particles and the thick terrestrial atmosphere. Variations of the UV ray intensity are predominantly controlled by cloudiness and depend on the season but not on the solar cycle variability. X rays are mostly absorbed by the atmosphere. A remaining echo of solar storms is mainly reflected in (i) climate changes at long temporal scales from years to centuries, (ii) changes in the atmosphere–ionosphere system at short scales, (ii) bio-effective variations of the geomagnetic field at various temporal scales (24), and (iv) variations of secondary cosmic rays born in the terrestrial atmosphere (29, 34).

Although cosmic rays contribute minimally to the energy input in the atmosphere, accounting for approximately 10^−9^ of the solar constant, they can affect human health in different ways. A direct effect of GCRs and SCRs suggests a random hitting of the subject, for example during taking a flight happened to coincide with GLEs, causing damage to DNA. Indirect effects are mainly associated with (i) ozone layer depletion caused by CRs and subsequent increasing in the carcinogenic UV radiation that reaches the ground in clear days; (ii) atmospheric ionization caused by simultaneous impact of CR, UV and X rays on the atmosphere, as shown in the bottom left sketch in Figure 6. When cosmic rays collide with atoms in the atmosphere, they initiate a series of reactions known as the “cosmic ray cascade.” This cascade starts with high-energy particles colliding with nuclei in the upper atmosphere, triggering what is called a spallation reaction. This reaction involves a highly energetic nucleon, often a secondary cosmic-ray neutron, colliding with a target nucleus. As a result, multiple particles – protons, neutrons, and clusters, are released. These particles instigate a chain of secondary interactions and further spallation reactions. As they accelerate, they continue striking atmospheric nuclei, generating additional particles and high-energy radiation. While these particles maintain their trajectory, photons are emitted in all directions, resulting in a net loss of energy to the atmosphere.

The main effects of CRs on the atmosphere occur above the altitude where airplanes fly (49), but the lower atmosphere can also be sensitive to CRs via atmospheric electricity, and a complex tie between CRs and geomagnetic variations (50–53). The main consequences of ionization of the atmosphere are shown in the scheme in the bottom right panel in Figure 6. CRs ionize molecules in the atmosphere, primarily nitrogen and oxygen, creating ions and free electrons. These ions can initiate chemical reactions involving atmospheric constituents. Ionization by CRs leads to the formation of free radicals from atmospheric molecules. Free radicals are highly reactive species and can participate in numerous atmospheric chemical reactions. Importantly, they are involved in oxidative stress (54). CRs can also affect aerosol formation by ionizing molecules, which can lead to the aggregation of the ions and their subsequent growth into aerosol particles. These aerosols can serve as sites for heterogeneous reactions involving pollutant gasses. Free radicals generated by CRs can participate in chemical reactions in the atmosphere. They might react with pollutants like nitrogen oxides (NOx) or volatile organic compounds (VOCs) through processes like oxidation or photolysis. CRs may also play a role in the formation and destruction of ozone (O3) in the atmosphere. Ozone formation occurs through the interaction of oxygen molecules with UV radiation, where cosmic rays may indirectly influence this process.

Analyzing variations in the CR flux allows us to take into account direct and indirect effects of space weather reflected in data from neutron monitors. If the Sun is quiet, neutron monitors measure a background level determined by galactic cosmic rays sometimes modulated by large-scale flows and streams propagating in the solar wind, but quickly after flares, the intensity of secondary cosmic rays increases considerably. These sporadic GLE events make huge diversity in cosmic ray data, and the corresponding changes in space weather may represent a danger not only for astronauts or pilots but also for creatures living on the Earth.

Additionally to a multi-year CR data analysis performed below for two particular places, we made a simple check on possible relation of the leukemia incidence with other space weather parameters that are typically used in such kinds of studies. So-called Wolf numbers (or the sunspot number) follow the course of the 11-year cycle of solar activity and are often used in studies of a possible impact of space weather on the biosphere without identification of a certain mechanism affecting bio-objects because it is unclear what physical agent or a combination of agents can cause a particular disease. It is important to stress that sunspot numbers just reflect the course of the solar cycle and cannot be considered in medical studies as an agent affecting human health. Finding a high correlation with the Kp index, described in Materials and methods, in turn, more definitely points out a relation of the process to bio-effective variations in the geomagnetic field because the planetary Kp index reflects the intensity of geomagnetic storms. We have checked both parameters and found that in the case of childhood leukemia there is no significant correlation between the sunspot number and the leukemia incidence rate found. r = −0.3 for Canada, r = −0.2 for the USA and r = −0.4 for Russia. The cross-correlation analysis shows that a curve shift does not improve the result. As for the correlation with the Kp index of the geomagnetic activity, it is absent for Canada, while r = −0.3 for the USA, and is the largest for Russia (r = −0.7).

#### 3.3.2 Cosmic rays as a possible factor shaping variations in the leukemia incidence

As mentioned above, a correct way to analyze variations of cosmic rays and their possible association with leukemia incidence is to choose cosmic ray stations nearby a location where leukemia statistics is gathered. A comparison of cosmic ray data with the whole-country data on any disease does not make sense because the intensity of cosmic rays at the ground level strongly depends on atmospheric factors in a certain place; therefore data from single neutron monitor stations near the location of the medical experiment should be used. We show below the data from the Newark (US) and Moscow (Russia) CR stations. The corresponding data for childhood leukemia are from New Jersey and Moscow with suburbs comparable by the area with New Jersey.

**Figure 7:**
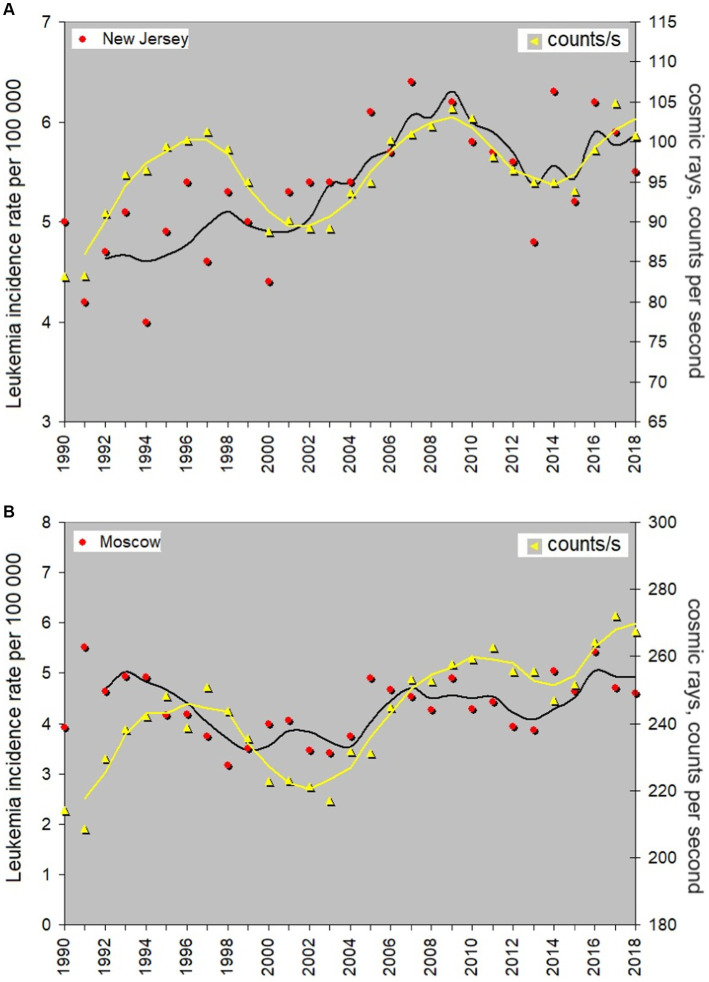
Annual average incidence rate of childhood leukemia in New Jersey and Moscow, exploring its potential correlation with the adverse effects of cosmic rays. Red dots are data for the leukemia rate and yellow triangles are counts per second of the secondary cosmic rays impacting neutron monitors. 3-year moving average is shown by yellow for cosmic rays and black for the leukemia rate.

Figure 7 illustrates a possible provocation of the development of leukemia in children by space weather effects reflected in enhancements in the cosmic ray intensity. Data for single cities are always more variable than the data averaged for the whole country, but it is easy to see that the general trend is also uprising, similar to that seen in smoother data averaged over a country. The question arises: Are these variations random or are they determined by some environmental processes? In the particular case shown in Figure 7, variations in the CR flux seem to modulate the leukemia incidence rate. To illustrate that we show the running average curves for both data rows. The corresponding correlation coefficients for Moscow and New Jersey are 0.42 and 0.56, respectively. Leukemia incidence begins to rise at the early stage of the cosmic ray flux increase related to solar activity. One may conclude that the variations in the cosmic ray intensity may be a reason for the variability in the leukemia incidence rate, suggesting direct or indirect mechanisms of impact.

We have tested possible links of the disease rate with local characteristics of the atmosphere that may be impacted by the CR flux variations. No relation of the leukemia incidence rate to the atmospheric pressure, humidity, temperature and the UV index is found in Moscow and New Jersey, but the comparison of the disease curve with ozone concentration gives a satisfactory result. One can see the corresponding information in Supplementary Figure S9. It is not easy to find open-access data for ozone in different countries, and checking this hypothesis requires gathering a material from local data archives. We just show one related graph using open-access data for New Jersey, US (the same location as used in the analysis shown in Figure 7), that allows suggesting the existence of such a link; however, a further investigation is required.

#### 3.3.3 Protective effect of the Earth’s magnetic field and consequences of loosing this protection as reflected in global leukemia statistics

The CR flux that reaches the ground mainly depends on (i) the height above the sea level, (ii) the volume of atmospheric mass above the particular place, and (iii) the geomagnetic latitude and intensity of the geomagnetic field. The combination of (ii) and (iii) leads to the situation when, generally, CRs are more dangerous closer to the poles. Indeed, the terrestrial atmosphere is thinner at high latitudes and thicker at low latitudes; this prevents energetic particles to reach the ground at low latitudes but makes high-latitude countries more vulnerable. CRs are charged particles and, therefore, they follow magnetic field lines, propagating along them to cusps near the poles of the geomagnetic field, shown in the upper panel of Figure 6.

A general comprehension of the global picture suggests that if the carcinogenic effect of CRs exists, it may be noted in the countries located closer or within the auroral ovals, the footprints of the cusps where aurora borealis and aurora australis are seen. Indeed, such countries show some increase in the childhood leukemia incidence rate in comparison with others, but as we noted in the introduction, the most suffering countries are located in South America.

**Figure 8:**
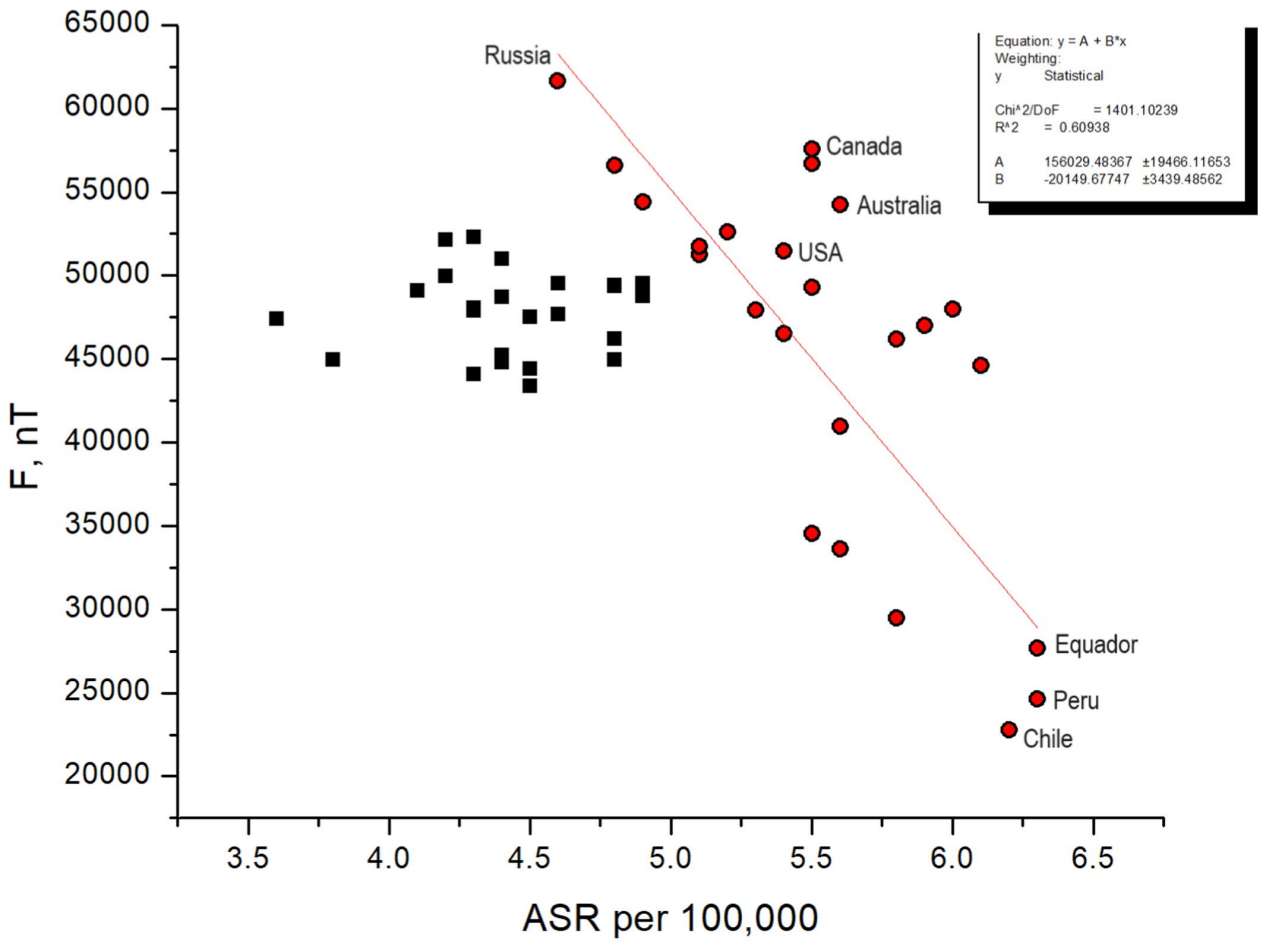
ASR statistics for 49 countries vs. intensity of the total magnetic field of the Earth. Countries situated in regions with intense geomagnetic field near the polar ovals, as well as those positioned in areas aligning with the dip in the geomagnetic field, experience the greatest impact from CRs. ASR statistics for such countries (red dots) generally follows the red line with parameters shown in the box.

To understand a reason for that, let us compare the total Earth’s magnetic field intensity *F* as calculated for 49 countries participating in the collection of childhood leukemia statistics from the International Agency for Research on Cancer with the corresponding ASR values for each country (see the original ASR data following the link in the Appendix, the data can also be found in Supplementary Table S4). The result is shown in Figure 8. The intensity of the magnetic field at the country level is estimated via the geomagnetic field calculator https://www.ngdc.noaa.gov/geomag/calculators/magcalc.shtml?useFullSite=true#igrfwmm. *F* is impacted by space weather and its values vary across the country, meanwhile the values shown in Figure 8 reflect a typical level of the geomagnetic field intensity with the accuracy at least to the first two digits. The best fit for Figure 8 is the fifth order polynomial with coefficients shown in Supplementary Figure S10. The four countries participating in the trend analysis shown above in Figures 2, 3, 5 are indicated next to the corresponding points in Figure 8.

The first feature that catches the eye is that the highest ASRs correspond to the lowest *F* values. One can find that the countries fall into two distinct categories – those with a high ASR level, exceeding 5 per 100,000, with a trend generally following the red line (the countries marked by red dots), and those associated with the middle intensity magnetic field with typical ASRs below 5 per 100,000 forming a cloud of black squares in Figure 8. The 24 countries forming the first category are Peru, Equador, Chile, Malta, Croatia, Italy, Colombia, Cyprus, Philippines, Costa Rica, Australia, New Zealand, USA Hawaii, Canada, Germany, Greece, USA, Switzerland, Sweden, Lithuania, Norway, China, Ukraine and Russia. The 25 countries from the second category are Austria, Belgium, Netherlands, Lebanon, Ireland, Spain, Czech Republic, UK, France, Thailand, Portugal, Japan, Belarus, Jordan, Hungary, Kuwait, Bahrain, Bulgaria, Slovenia, Iceland, Estonia, Poland, Slovakia, Israel, and France. Note that the segregation is approximate, and there may be intersecting entities.

**Figure 9:**
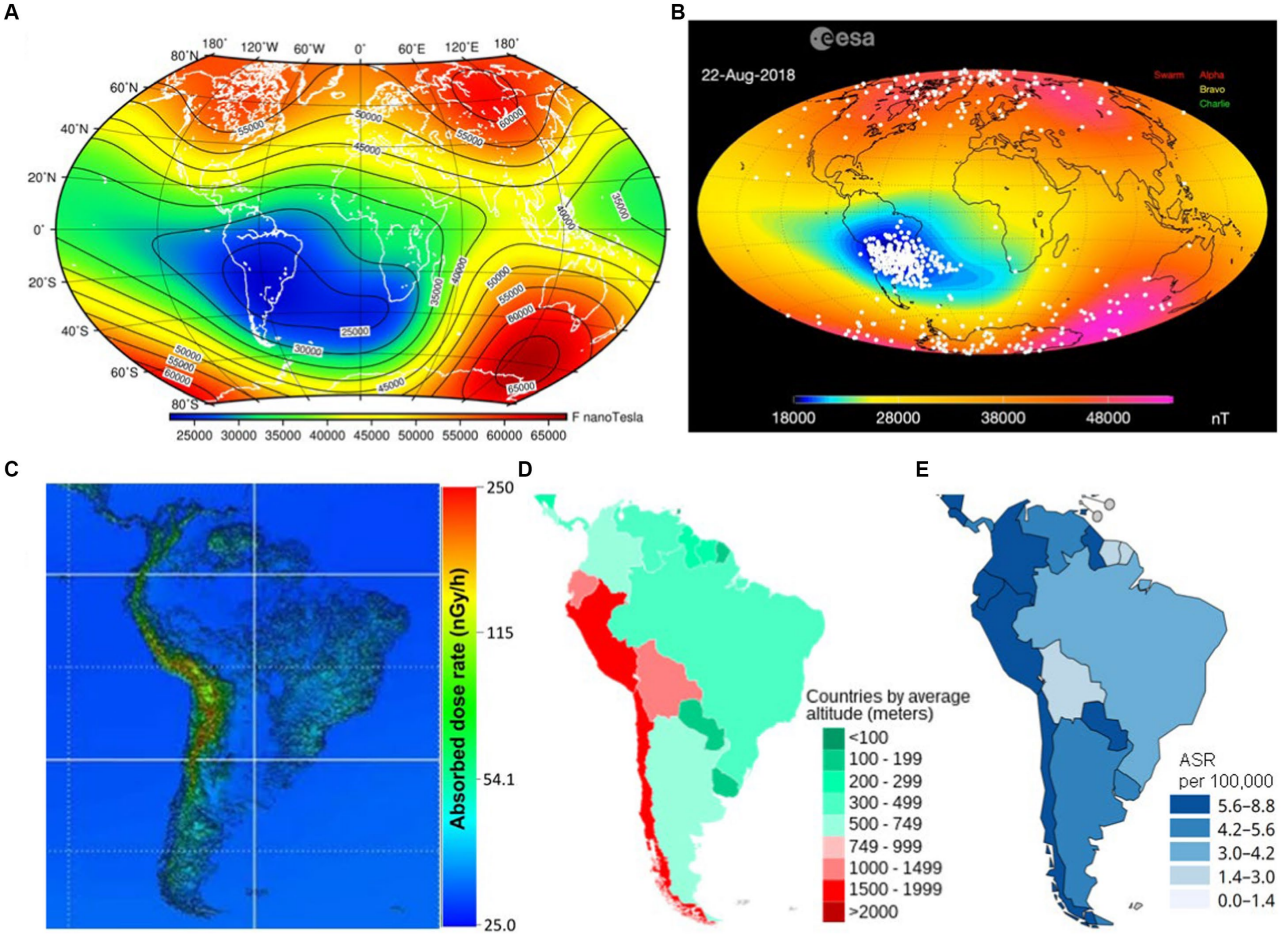
Spatial distribution of the terrestrial magnetic field and CR flux intensity that may govern the leukemia incidence in different locations. **(A)** Total magnetic field intensity equipotentials. Red corresponds to the strongest geomagnetic field, and deep blue denotes the weakest geomagnetic field. Countries located at the same geographic latitudes may be characterizes absolutely different magnetic field intensity levels. **(B)** Snapshot of the ESA movie showing the observed impact of energetic particles and CRs over the globe. CRs follow magnetic field lines, therefore they precipitate mainly over auroral zones because this is close to magnetic poles (see Figure 6) and where South Atlantic Anomaly is located (blue spot). **(C)** Modeled absorbed CR dose rate in South America [adapted from Sato (55)]. South Atlantic Anomaly impact is not taken into account. **(D)** Average elevation of South American countries (data provided by US Central Intelligence Agency). **(E)** Fragment of Figure 1. Impact of CRs on people strongly depends on height. Elevated countries are at increased radiation risk everywhere, especially in the area of South Atlantic Anomaly.

The geographic location of the courtiers has rather poor association with the typical characteristics of the geomagnetic field; as illustrated in Figure 9A (see also http://www.geomag.bgs.ac.uk/education/earthmag.html), therefore one should not be surprised to find that two countries located at very different geographic latitudes have the same *F*. Therefore, knowing the information from 3.3.1, we can interpret Figure 8 in the way that the countries with the middle *F* values corresponding to the middle geomagnetic latitudes are most protected from CRs, but the countries located closer to auroral ovals, both in Northern and Southern hemispheres, with larger *F* suffer from childhood leukemia in a higher degree because CRs propagate along magnetic field lines and precipitate in auroral ovals expanding considerably during geomagnetic storms.

An important point is that the largest ASR values correspond to the countries located in the vast area called South Atlantic Anomaly, which represents a deep hole in the terrestrial magnetic field into which energetic particles and cosmic rays fall (56). It is known as the most dangerous spot for satellites and airplanes in terms of the radiation hazard, even more dangerous than auroral ovals. Figure 9B is a snapshot of an observational picture of energetic particle precipitation over the globe as observed by the SWARM probe. One can find the corresponding video and the explanation of the problem on the European Space Agency (ESA) educational website: https://www.esa.int/Applications/Observing_the_Earth/FutureEO/Swarm/Swarm_probes_weakening_of_Earth_s_magnetic_field. Therefore, all countries lying within the anomaly or close to it experience unprecedented impact of CRs, and the thick atmosphere at lower latitudes does not help in the particular case because South Atlantic Anomaly represents a bottle-like magnetic trap for high-energy charged particles (57).

Furthermore, South America is characterized by a high averaged altitude, which leads to increased absorbed dose of CR radiation at the ground level as shown in Figure 9C adapted from Sato (55). The dose is calculated on the basis of an analytical CR flux model (58) that takes into account the elevation of the particular place illustrated in Figure 9D and employs the dipole magnetic field approximation, which means that South Atlantic Anomaly is not taken into account. Despite that, it is easy to see that the CR dose rate and the country elevation map show a striking similarity with Figure 1 for ASR in South America (the corresponding fragment of Figure 1 is extracted to Figure 9E). One may suppose that, according to Figure 9B, the presence of South Atlantic Anomaly worsens the situation in comparison with that predicted in Figure 9C, but calculating the actual CR flux dose using all realistic peculiarities is a resource-consuming task which has not been performed yet.

Now one can explain why ASR statistics for South African countries shows dramatically high disease rates. In terms of the radiation hazard, both the countries to which auroral ovals extend and the countries located in the South Atlantic Anomaly zone are associated with a higher risk of childhood leukemia in comparison with the countries marked by black squares in Figure 8 that are protected by both rather thick atmosphere and rather strong geomagnetic field shaped like a cover above them. We specify the three most affected countries in Figure 8 to stress the fact that their locations are associated with extremely low level of the magnetic field and a bottle-like-shaped magnetic field dip, therefore the protection against CRs is lost there.

## 4 Conclusions and discussion

This study aims to draw an attention of both the medical and physical communities to the unresolved issue of identifying environmental triggers and drivers responsible for childhood leukemia. The introduction highlights key factors implicated in childhood leukemia that can be divided into environmental and non-environmental categories. The latter category includes genetic predisposition, encompassing mutations (like those seen in the Down syndrome and Li-Fraumeni syndrome cases) and immune system disorders, which compromise the body’s ability to combat abnormal cells. Previous chemotherapy and radiation treatments are known to elevate childhood leukemia risk, particularly due to potential post-treatment complications. Additionally, age and gender play roles, with leukemia more prevalent in young children, and variations in leukemia types between genders. There are ethnic disparities, with certain groups like Hispanic children exhibiting higher leukemia rates, likely influenced by genetic, environmental, and socio-economic factors. Having a family history of leukemia, particularly in a first-degree relative, is also associated with an increased risk of developing the disease. Subtypes of leukemia may be linked with racial differences. This suggests either a potential genetic predisposition to leukemia within certain families or consequences of family diet or lifestyle habits that sometimes are kept for generations (59, 60).

Environmental factors include exposures to ionizing radiation and chemicals/gasses that may contribute to leukemia development. This may include direct effects of cosmic rays, ozone and radon (61–63). The latter risks may intersect with certain lifestyle and even ethnic factors mentioned above, as the ozone concentration level is generally larger in the countryside and radon particularly poses a threat to individuals residing in basements or on lower floors of buildings with cracks in floors and walls, or inadequate ventilation. Additionally, some studies indicate a potential association between exposure to electromagnetic fields, such as those emitted by power lines or electrical appliances, and childhood leukemia.

The distinction between non-environmental and environmental factors lies in their correlation patterns: the former do not generally correlate and vary across different locations, forming the foundation of statistical distributions of leukemia rates. In contrast, the latter can simultaneously vary across much larger areas, potentially resulting in noticeable variations and trends in leukemia rates at the country level.

We have analyzed annual variations in the incidence of childhood leukemia in the USA, Canada, Australia and Russia and linked the trends found with statistics for the vehicle exhaust gasses and smoke from fires in the corresponding countries. Unique data collected from all regions of Russia for 1990–2018 are shown for the first time. We have also expressed a hypothesis about possible space weather effects both at the local level, showing a link between the CR flux and the disease rate long-term variations in two particular places where it is possible to measure the CR directly. We have analyzed general childhood leukemia statistics data for 49 countries with respect to the geomagnetic field parameters responsible for CR precipitation.

Our results on the long-term trend comparison confirm previously found tendencies for the childhood leukemia incidence growth known for the USA, Canada and Australia (40–42) and reveal for the first time that the number of children suffering from leukemia in Russia is growing twice as fast as in the US and Canada. The study confirms a strong impact of toxic gasses from cars and fires on children, previously found in many works (e.g., 16, 24, 25), with a primary role of gasses from transport.

We have shown that CR flux absorbed dose maps generally correspond to the world distribution of leukemia cases, and the carcinogenic impact of CRs widely discussed in the literature (e.g., 29, 33–36, 64) may be especially strong in the areas associated with South Atlantic magnetic field Anomaly representing a hole in the terrestrial magnetosphere, which may cause a known peak in the disease rate in this region.

The childhood leukemia incidence rate correlates with the number of cars owned by citizens of all three countries the best, up to the level of 0.9, which perfectly describes the observed large-scale increasing trend and makes carcinogenic pollutants from cars the most dangerous for children’s health. The other potentially dangerous physical agents are cosmic rays and bio-effective variations in the geomagnetic field. The study suggests that parents-to-be may be exposed even before giving birth to their children. Another reason may be that there is a delay in the development of leukemia in children. One can expect the leukemia incidence rate in the USA, Australia and Canada decrease since benzene cars are actively replaced with electric and hybrid cars in these countries. In Russia, on the contrary, the situation is expected to worsen because electric and hybrid cars are practically not used there and the number of benzene cars grows rapidly.

Leukemia is considered as a treatable disease and the survival rate in developed countries increases year by year. Meanwhile, knowing what environmental factors provoke its start is crucial for prevention of the disease. This study shows that the leukemia incidence rate steadily rises in all three urban countries at the large time span of almost 30 years, which cannot be just a natural process but is most probably related to human activities and a style of life in developed countries. First of all, finding the monotonous growth trend common for all three countries allows us to assume the presence of common causes of anthropo-technogenic nature. Gasses from cars seem to be the most dangerous factor affecting children. Toxic gasses from fires may also impact the children’s health negatively, with some delay that suggests a possible impact on future parents too (38, 65). Indeed, based on the results of prior studies, the factor that constantly increases its influence with the development of civilization and is most likely involved in the increase in the incidence of leukemia in children is air pollution. All types of smoke, including tobacco, wood, coal and others, contain polycyclic aromatic hydrocarbons, dioxins, carbon microparticles PM10 and PM2.5, and therefore are carcinogenic to humans (66).

Of particular note are the exhaust gasses of transport, which differ from other types of smoke in the presence of benzene. In accordance with the gradation of the International Agency for Research on Cancer, benzene is assigned to group 1 and is a proven human carcinogen (66). During the combustion of benzene, benzpyrene is formed. According to the degree of toxicity, it also belongs to the first hazard class gas. It has been found that the exposure of an expectant mother to gasoline, aromatic hydrocarbons and exhaust gasses significantly increases the likelihood of developing leukemia in her offspring (67). In Russia, in 1997–2018 there was a threefold increase in the number of passenger cars per 1,000 population (see Supplementary Table S3). In the United States and Canada, the car park is currently more than two times larger than the Russian level and continues to grow. Based on the foregoing, the hypothesis that the increase in the frequency of childhood leukemia is mostly associated with vehicle smoke seems to be the most reasonable.

The leukemia incidence rate in Russia is lower than in the other countries but it grows twice faster. This unfortunate situation is, first of all, because of the delay in the transport development in Russia in comparison with the other countries. We do not know statistics for childhood leukemia in the USA, Australia and Canada in 1950–1980th to compare with the current state of this disease in Russia but the data for transport are known and show similar trends for the after-WWII-period in the US and in modern Russia. Similar ideas can be found in the press-release: https://www.energy.gov/eere/vehicles/fact-962-january-30-2017-vehicles-capita-other-regionscountries-compared-united-states/. Therefore, current trends in childhood leukemia seen in Russia may give us an idea about the unknown past level of this disease in the other urban countries.

On the other hand, the effect of the super-growing leukemia rate in Russia may be determined by differences in approaches to saving ecology and measures taken for cancer prevention. The content of benzene in gasoline in the USA is 1–5%, in Europe 3–5% (25). In Russia, the amount of benzene in gasoline has been limited to 5% by volume too. However, recently the Russian government, following the economic decline, has announced plans to completely lift all ecological limitations and allow using engines of the so-called Euro-0 class on the roads. According to the results of our study, this will lead to an abrupt increase in the childhood leukemia rate in a very short perspective since the Russian residents are unevenly distributed across the country and mostly live in big cities in which the car number is the highest. Taking into account the whole unfortunate situation in the country and the fact that the rate of similar diseases also increases year by year and, if the trend continues, this will dramatically affect the whole population. The estimation of the resulting increase in the death rate at the population level is our next goal. Meanwhile, it is expected that the leukemia incidence rate in the USA, Australia and Canada may decrease soon because of active usage of electric and hybrid cars in these counties.

One may suggest that the risk of leukemia in children depends on a combination of acting agents, overlapping effects of which increase a threshold. In other words, the attempts to find a solo cause of childhood leukemia fail because the effect is collective and only exceeding the threshold leads to the development of the disease. If so, further studies are required to build models out of so-far-known factors that may cause leukemia. The models must consider overarching trends, as shown in Figures 3, 5, as well as localized impacts, like those shown in Figure 7 and Supplementary Figure S9. In parallel, it is important to study certain mechanisms behind the associations found in the study. A correlation analysis or an analysis of trends never confirms the direct action of one parameter on the other, it merely suggests potential pathways for understanding the physical and chemical processes depicted in the figures. One of important directions is to study how rather well-known processes go in extremely polluted atmospheres of big cities.

An increased pollution in urban countries leads to a formation of unnaturally composed atmosphere around big cities, and possible consequences of the impact of cosmic rays on such an atmosphere are poorly known. For instance, chemical reactions shown in Figure 6 may lead to ozone destruction in the atmospheres of big cities, analogous to that happening to the ozone layer. According to Supplementary Figure S9, ozone may be one of the factors directly impacting children. Note that the correlation coefficient is negative, which means that less ozone in the urban area is associated with more cases of childhood leukemia. An association of ozone with non-respiratory cancers is unclear. Ozone at the ground level is formed due to solar radiation affecting airborne pollutants, primarily nitrogen oxides and volatile organic compounds. Traffic is a commonly known source of ozone in big cities. It is important to note that both excessively low and excessively high levels of ozone concentration can be detrimental to living organisms. It is known that the ozone concentration is generally higher in rural areas than in cites, which is called the “ozone paradox”.^4^ The relationship between atmosphere pollution in big cities and ozone production is not straightforward and should be studied in future to understand its possible impact on the development of childhood leukemia.

The other not straightforward association of childhood leukemia with variations in cosmic rays, known as a cancer-associated factor, presents a complex and not entirely understood connection. While the precise biophysical processes responsible for this association remain elusive, it is postulated that the cumulative effect of radiation exposure may play a significant role. This hypothesis suggests that exceeding a certain absorbed dose threshold could be pivotal, with potential variations in threshold levels, particularly notable for pregnant women compared to non-pregnant women. For example, one may suppose that additional exposure of any kind like taking a flight while being pregnant is a risk factor for women from South America, although there are no such studies carried out.

Our work underscores the heightened radiation risk observed in countries situated (i) in regions with diminished geomagnetic field intensity, (ii) within the auroral oval zones although characterized by an increased intensity of the geomagnetic field, but, structurally, representing large dips in the terrestrial magnetic field that allow energetic particles to enter the atmosphere, and (iii) in South America. South America’s elevated terrain and the presence of the South Atlantic Anomaly (hole) in the geomagnetic field exacerbate this risk, as this region, along with (i) and (ii), experiences compromised protection from cosmic rays provided by the geomagnetic field. These findings highlight the importance of further research into the biophysical mechanisms underlying the association between childhood leukemia and cosmic ray variations. Additionally, they underscore the necessity for targeted public health interventions and policies aimed at mitigating radiation risks, particularly in vulnerable populations such as pregnant women and children.

**Figure 10:**
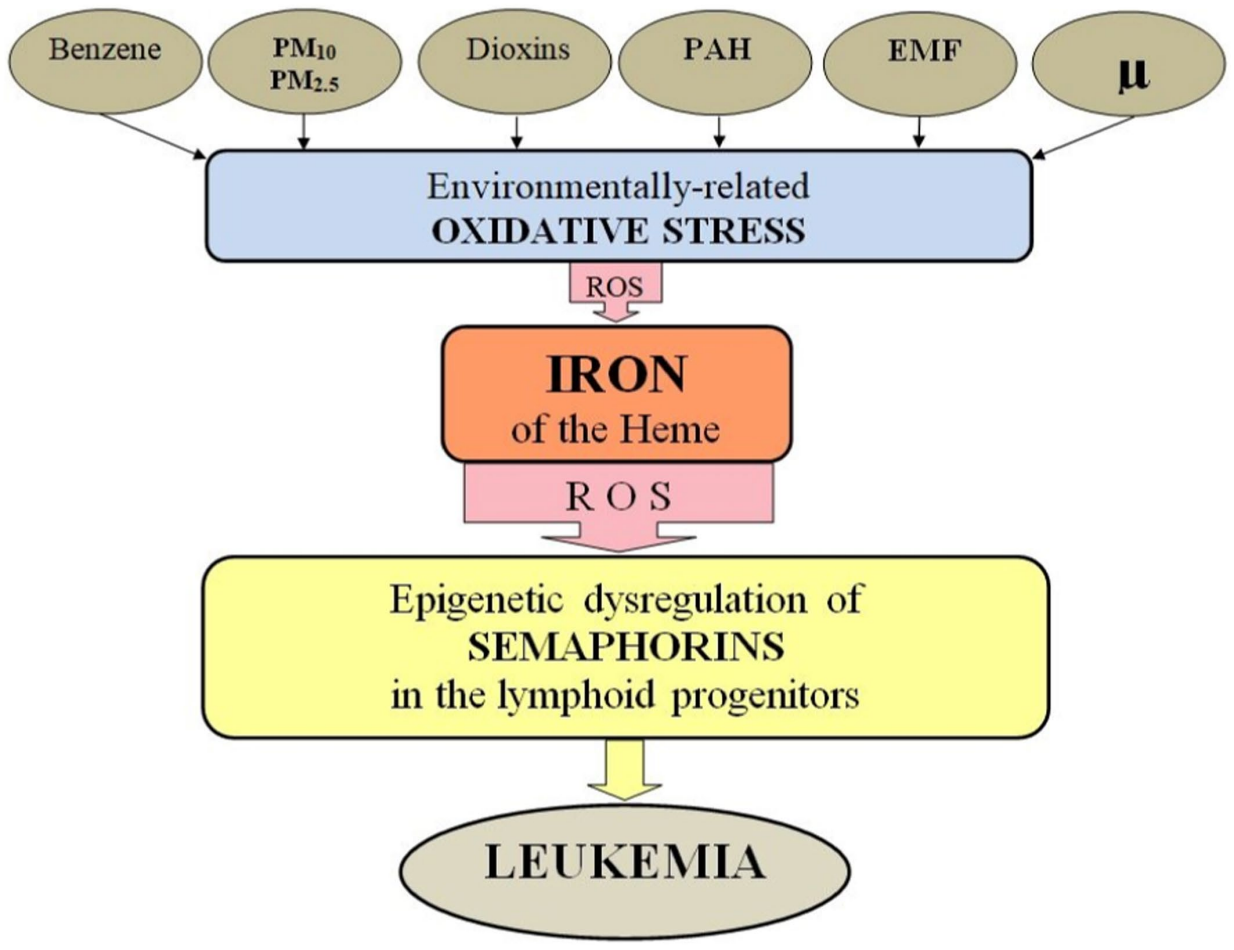
Scheme of environmentally-related leukemogenesis. Carcinogenic environmental factors cause environmentally determined oxidative stress, which is enhanced by heme iron. This leads to epigenetic dysregulation of semaphorins in lymphoid progenitors and the onset of leukemia. PM10 and PM2,5, fine atmospheric particles with a diameter of 10 μm and 2.5 μm; PAH, polycyclic aromatic hydrocarbon; EMF, electromagnetic fields; μ, secondary cosmic rays; ROS, reactive oxygen species.

The following scheme for the occurrence of leukemia in children, in which oxidative stress plays an initial role, can be proposed (see Figure 10). Benzene, carbon microparticles, dioxins, polycyclic aromatic hydrocarbons (68), electromagnetic waves of bio-effective frequencies (34, 69), and cosmic radiation (70) acting altogether induce the stress. After that, the oxidative stress is enhanced by heme, which, due to chemical instability, triggers the cyclic “ROS → Heme→ROS” chain reaction (71). It leads to an avalanche-like increase in the number of formed aggressive radicals. The heme of the embryo, fetus, and newborn is most susceptible to these processes (53). Additionally, due to the ferromagnetic properties of heme iron, the reception, induction and possible amplification of the variable electromagnetic field occur with the formation of a feedback loop and even possible greater stimulation of oxidative stress (71). In areas of high heme concentration, in particular in the bone marrow, oxidative stress reaches its maximum and leads to modulation of the epigenetic apparatus at all levels (72). The occurrence of such disorders in the regulation of semaphorins SEMA3A and SEMA4D contributes to the development of leukemia (73). More studies are required to confirm or reject this hypothesis. Character μ (for muons) refers to the scheme shown in Figure 6.

Long-term changes in the ASR curve course may be determined by some major factors acting over large territories, slowly rising and affecting children. Meanwhile, relatively short-term variations of the disease curve may be associated with local environmental changes.

In sum, the results of our study support the idea of a major role of anthropogenic pollutants in causing childhood leukemia. A dramatically fast growth of the leukemia rate in Russia in comparison with that observed in the USA, Australia and Canada can partially be explained by its later urbanization and poor pollution prevention. Our findings highlight that the most dangerous pollutants are carcinogenic gasses emitted from both cars and fires, which determine the main course of the leukemia incidence rate in all countries. There are also signatures of the influence of space weather reflected in cosmic ray variations on the childhood leukemia development. Since the impact of cosmic ray variations is local and depends on the altitude and geomagnetic latitude of the place of living, more studies are required to understand the risk of the childhood leukemia development caused by cosmic ray intensity variations in particular countries. Overall, we suggest that the collective impact of all factors considered in the study may lead to the stress reaction that exceeds an ability of restoration of a young organism and causes leukemia.

## Data availability statement

The original contributions presented in the study are included in the article/Supplementary material, further inquiries can be directed to the corresponding author.

## Author contributions

OK: Conceptualization, Data curation, Formal analysis, Funding acquisition, Investigation, Methodology, Supervision, Validation, Writing – original draft, Writing – review & editing, Visualization. SP: Conceptualization, Data curation, Formal analysis, Funding acquisition, Investigation, Methodology, Supervision, Validation, Writing – original draft, Writing – review & editing. VC: Conceptualization, Data curation, Supervision, Writing – review & editing. AC: Conceptualization, Data curation, Supervision, Validation, Writing – review & editing. OP: Supervision, Writing – review & editing.

## Footnotes

^1^ https://vizhub.healthdata.org/gbd-compare/#

^2^ http://www.oncology.ru/service/statistics/malignant_tumors

^3^ https://nodgo.org

^4^ https://www.irceline.be/en/documentation/faq/why-are-ozone-concentrations-higher-in-rural-areas-than-in-cities

## Appendix

The datasets for Russia used for this study can be found in the Supplementary material S1. Data on the childhood leukemia incidence in Russia are collected from annual reports of the P. Hertsen Moscow Oncology Research Institute (https://nmicr.ru/en/about/). The data for some years can be found on the informational website of the Federal State Budgetary Institution “NMRRC” of the Ministry of Health of Russia http://www.oncology.ru/service/statistics/malignant_tumors. One should register first on the main page to get an access. Analogous data for the USA, Canada and Australia are available on https://seer.cancer.gov/statistics-network/, https://www150.statcan.gc.ca/t1/tbl1/en/tv.action?pid=1310011101, and https://cancerqld.org.au/research/queensland-cancer-statistics/accr/, respectively. Incidence rate of Childhood Leukemia in New Jersey is taken from the public New Jersey’s Public Health Data Resource https://www-doh.state.nj.us/doh-shad/indicator/complete_profile/LeukemiaChild.html?ListCategoryFirst=x. Averaged childhood leukemia incidence rate in different countries used for the comparative study is taken from the International Agency for Research on Cancer https://iicc.iarc.fr/results/comparative-tables/.

Children population and total population census data for Russia listed in the Supplementary material S1 are provided on Russian Federal State Statistics Service (Rosstat) website https://rosstat.gov.ru/folder/12781. Children population for other countries can be found in the United Nations Population Division’s World Population Prospects, 2022 Revision https://api.worldbank.org/v2/en/indicator/SP.POP.0014.TO?downloadformat=excel. Note that the latter data is estimation and may slightly vary from the corresponding data on national resources. Population census data for the USA, Canada, Russia, and Australia are from Microtrends and United Nations – World Population Prospects (see https://www.macrotrends.net/countries/).

Wildfire and deforestation data for Canada, the USA, and Russia can be found in the National Forestry Databases http://nfdp.ccfm.org/en/data/fires.php, https://www.nifc.gov/fire-information/statistics/wildfires, and https://ierarp.ru/ploshhad-lesnyx-pozharov-v-rossii-s-1990-po-2018-god/, respectively. The Australia area burnt data from Landgate NOAA-AVHRR is provided by McNeely et al. (37) and can be found in the “Multi-decadal increase of forest burned area in Australia is linked to climate change.V1.2.xlsx” file on https://zenodo.org/records/5631766.

Data for the vehicle number are from Rosstat for Russia (https://rosstat.gov.ru/statistics/transport). The number of vehicles in use in the USA are from the Federal Highway Administration of the US Department of Transportation, https://www.google.com/publicdata/explore?ds=gb66jodhlsaab_, and the normalized data are available in the table https://tedb.ornl.gov/wp-content/uploads/2022/03/Table3_08_01312022.xlsx from the Transportation Energy Data Book Edition 40 provided by the Oak Ridge National Laboratory of the US Department of Energy https://tedb.ornl.gov/data/. Analogous data for Canada can be found at https://www150.statcan.gc.ca/t1/tbl1/en/tv.action?pid=2310006701, and for Australia – from the Australian Bureau of Statistics https://www.abs.gov.au/statistics/industry/tourism-and-transport/motor-vehicle-census-australia. Note that the data for Canada are available from 1999 only (see https://www23.statcan.gc.ca/imdb/p2SV.pl?Function=getInstanceList&Id=1529932).

Data on the sunspot number and Kp index are from the official Goddard Space Flight Center OMNIweb database https://omniweb.gsfc.nasa.gov.The Neutron Monitor Database provides data for secondary cosmic rays detected at the ground level (www.nmdb.eu). Magnetic field of the Earth is calculated using the most recent World Magnetic Model provided by NOAA https://www.ngdc.noaa.gov/geomag/calculators/magcalc.shtml?useFullSite=true#igrfwmm.
