# Supplementary material for: Trends in childhood leukemia incidence in urban countries and their relation to environmental factors, including space weather
Source: Front Public Health. 2024 May 2;12:1295643. doi: 10.3389/fpubh.2024.1295643 (PMC11098134; doi:10.3389/fpubh.2024.1295643)
Supplement: Supplementary file 1 [file Data_Sheet_1.doc]

Supplementary Material

# Trends of Childhood Leukemia in Urban Countries and Their Possible Relation to Environmental Factors, Including Space Weather

Olga Khabarova*, Sergey K. Pinaev, Vladimir V. Chakov, Alexey Ya. Chizhov, Olga G. Pinaeva

*** Correspondence:** Olga Khabarova: [olgakhabar@tauex.tau.ac.il](mailto:olgakhabar@tauex.tau.ac.il)

**1. Diagnostic Process**

The diagnosis of acute lymphoid leukemia in Russia is made out according to the standards of medical care in the Russian Federation posted on the official website of the National Society of Pediatric Hematologists and Oncologists, see <https://nodgo.org/> and references **(S1-S4)** below. It is established based on the detection of more than 25% of leukemic cells in the bone marrow aspirate, for which one of the variants of lymphoid differentiation of blast cells is proved using multiparametric flow cytometry and Cytochemistry data and the absence of the activity of myeloperoxidase and nonspecific esterase as key markers of non-lymphoid progenitor cells is shown. The diagnosis of acute myeloid leukemia is made when at least 20% of blast cells are found in the bone marrow punctate or in peripheral blood, or regardless of the percentage of blast cells in the presence of pathognomonic for AML chromosomal t(8; 21) (q22; q22) AML/ETO, t(15;17) (q12; q11-12) PML/RAR-α, inv(16) or t(16;16) (p12;q23) CBF/MYH11, t(1;22). The diagnosis of chronic myeloid leukemia is necessarily confirmed by detection of the Ph(+) chromosome during routine cytogenetic analysis, translocation t(9;22) (q34;q11.2), FISH method, or detection of RNA of the chimeric BCR/ABL1 gene by polymerase chain reaction. Acute promyelocytic leukemia in children is confirmed on the basis of cytological and cytochemical examination of bone marrow punctate and in all cases verified on the basis of cytogenetic and/or molecular genetic analysis with the detection of a specific translocation t(15; 17) and / or the presence of a specific transcript (PML/RARK). Data for 1990-2018 for Russia and Moscow are provided below in **Table S1**.

# 2. Supplementary Tables containing the data utilized in the investigation

For the readers’ convenience, we show below the data employed in the study. **Table S1** outlines the annual counts of childhood leukemia cases in Russia and its capital, along with population statistics spanning from 1990 to 2018. These data are referenced in Figures 2-5 and Figure 7 of the manuscript. **Table S2** details wildfire occurrences in the USA, Russia, Canada, and Australia, showcasing the annual fire areas recorded in these countries from 1990 to 2018. This information is utilized in Figure 3 of the manuscript. **Table S3** displays the yearly registration numbers of vehicles (excluding motorcycles) in the USA, Russia, and Australia from 1990 to 2018. Note that data for Canada are available only from 1999 to 2017, as presented in **Table S3**. This table contributes to the generation of Figure 5 in the manuscript. Lastly, **Table S4** presents the average incidence rates of childhood leukemia across 49 countries alongside the corresponding Earth's total magnetic field intensities in those regions. This data is referenced in Figure 8 of the manuscript. For the data sources, see the Appendix in the manuscript.

**Table S1.** Leukemia incidence (yearly number of new cases), child population (age 0–14, all races, both sexes), and total population in Russia and in Moscow. Total and child populations are calculated as the number of people on the first day of the subsequent year. Data for Crimea are not included.

| Year | New cases in children, Russia | New cases in children, Moscow | Child population, Russia | Child population, Moscow | Population – all ages , Russia | Population - all ages, Moscow |
| --- | --- | --- | --- | --- | --- | --- |
| 1990 | 825 | 66 | 33949361 | 1681663 | 148273746 | 9017415 |
| 1991 | 972 | 92 | 33670304 | 1670806 | 148514692 | 9067808 |
| 1992 | 937 | 76 | 33225630 | 1639308 | 148561694 | 9066025 |
| 1993 | 996 | 79 | 32568354 | 1600498 | 148355867 | 9066612 |
| 1994 | 1026 | 77 | 32049886 | 1566340 | 148459937 | 9085457 |
| 1995 | 956 | 64 | 31346286 | 1537433 | 148291638 | 9246727 |
| 1996 | 974 | 63 | 30518835 | 1507911 | 148028613 | 9411236 |
| 1997 | 888 | 55 | 29496739 | 1468587 | 147802133 | 9604297 |
| 1998 | 834 | 45 | 28315461 | 1419757 | 147539426 | 9783242 |
| 1999 | 791 | 48 | 27065804 | 1371307 | 146890128 | 9932932 |
| 2000 | 821 | 53 | 25916984 | 1328787 | 146303611 | 10114203 |
| 2001 | 776 | 52 | 24701597 | 1281636 | 145649334 | 10269900 |
| 2002 | 736 | 43 | 23553751 | 1243342 | 144963650 | 10386903 |
| 2003 | 779 | 43 | 22735321 | 1258327 | 144333586 | 10535681 |
| 2004 | 783 | 48 | 22103541 | 1281374 | 143801046 | 10726429 |
| 2005 | 787 | 63 | 21517789 | 1285528 | 143236582 | 10923762 |
| 2006 | 795 | 61 | 21194211 | 1303068 | 142862692 | 11091428 |
| 2007 | 771 | 60 | 21127315 | 1322425 | 142747535 | 11186851 |
| 2008 | 765 | 58 | 21345302 | 1355610 | 142737196 | 11281631 |
| 2009 | 859 | 68 | 21586863 | 1388788 | 142833502 | 11382161 |
| 2010 | 882 | 60 | 21769130 | 1396168 | 142865433 | 11776764 |
| 2011 | 841 | 65 | 22207236 | 1467893 | 143056383 | 11856578 |
| 2012 | 978 | 60 | 22817105 | 1522434 | 143347059 | 11979529 |
| 2013 | 956 | 61 | 23383808 | 1574144 | 143666931 | 12108257 |
| 2014 | 1019 | 82 | 24028347 | 1624742 | 146267288 | 12197596 |
| 2015 | 1037 | 78 | 24605684 | 1680251 | 146544710 | 12330126 |
| 2016 | 1100 | 94 | 25158466 | 1731345 | 146804372 | 12380664 |
| 2017 | 1179 | 84 | 25419120 | 1782217 | 146880432 | 12506468 |
| 2018 | 1187 | 84 | 25536578 | 1823888 | 146781096 | 12615279 |

**Table S2.** Area burnt in wildfires in the USA, Russia, Canada, and Australia by year.

| Year | Area burnt, hectares ×10^6^ –  USA | Area burnt, hectares ×10^6^ –Russia | Area burnt, hectares ×10^6^ –Canada | Area burnt, hectares  ×10^7^ –Australia |
| --- | --- | --- | --- | --- |
| 1990 | 1.870304 | 0.965000 | 0.953324 | 2.2354096 |
| 1991 | 1.195271 | 0.569000 | 1.545788 | 2.5070957 |
| 1992 | 0.837671 | 0.522000 | 0.851826 | 1.6336897 |
| 1993 | 0.727452 | 0.733000 | 1.950352 | 2.4126750 |
| 1994 | 1.648519 | 0.520000 | 6.161349 | 3.9488204 |
| 1995 | 0.744843 | 0.352000 | 7.375355 | 3.7744721 |
| 1996 | 2.454822 | 1.826000 | 1.861750 | 3.6199224 |
| 1997 | 1.156170 | 0.669000 | 0.632749 | 5.1461531 |
| 1998 | 0.538112 | 2.458000 | 4.741030 | 3.0513226 |
| 1999 | 2.276799 | 0.751700 | 1.717143 | 5.0786057 |
| 2000 | 2.992040 | 1.328600 | 0.634155 | 9.0237256 |
| 2001 | 1.445096 | 0.896800 | 0.647669 | 7.8326237 |
| 2002 | 2.907550 | 1.369500 | 2.763605 | 4.9925219 |
| 2003 | 1.602896 | 2.352800 | 2.168405 | 2.5988751 |
| 2004 | 3.277096 | 0.543300 | 3.182999 | 6.2165133 |
| 2005 | 3.516471 | 0.736300 | 1.686770 | 1.4228531 |
| 2006 | 3.995763 | 1.493500 | 2.100680 | 6.6858877 |
| 2007 | 3.774926 | 1.036100 | 1.785468 | 6.4631750 |
| 2008 | 2.141786 | 2.069800 | 1.664925 | 3.6790000 |
| 2009 | 2.396462 | 2.111600 | 0.762567 | 4.2555769 |
| 2010 | 1.385127 | 2.027800 | 3.177965 | 2.6830946 |
| 2011 | 3.525365 | 1.408400 | 2.397428 | 10.1179721 |
| 2012 | 3.774195 | 2.101200 | 1.811691 | 8.6550534 |
| 2013 | 1.748058 | 1.158000 | 4.268502 | 3.4091550 |
| 2014 | 1.455093 | 3.190700 | 4.545658 | 5.8207114 |
| 2015 | 4.097502 | 2.748900 | 3.908380 | 3.5132541 |
| 2016 | 2.229816 | 2.508300 | 1.319606 | 3.5493933 |
| 2017 | 4.057413 | 1.400000 | 3.589430 | 6.3620929 |
| 2018 | 3.548078 | 3.200000 | 2.328851 | 4.3995946 |

**Table S3. Vehicles registered (motorcycles are not included) in the USA, Russia, Canada, and Australia by year.**

| Year | Vehicles per Thousand People - USA | Vehicles per Thousand People - Russia | Vehicles per Thousand People-Canada | Vehicles per Thousand People - Australia |
| --- | --- | --- | --- | --- |
| 1990 | 773.4 | 59.6 |  |  |
| 1991 | 760.2 | 66.3 |  | 568.2 |
| 1992 | 758.0 | 73.0 |  |  |
| 1993 | 761.9 | 79.8 |  | 579.4 |
| 1994 | 766.9 | 86.5 |  |  |
| 1995 | 771.0 | 93.2 |  | 591.6 |
| 1996 | 781.2 | 100.8 |  | 600.8 |
| 1997 | 776.0 | 108.4 |  | 633.6 |
| 1998 | 781.2 | 116.0 |  | 648.7 |
| 1999 | 790.1 | 123.6 | 777.5 | 652.6 |
| 2000 | 800.3 | 130.5 | 770.0 |  |
| 2001 | 825.8 | 137.2 | 755.6 | 648.2 |
| 2002 | 815.7 | 145.8 | 772.6 | 658.4 |
| 2003 | 816.1 | 153.0 | 780.8 | 668.2 |
| 2004 | 829.9 | 158.9 | 789.6 | 679.2 |
| 2005 | 837.3 | 168.4 | 802.0 | 690.1 |
| 2006 | 840.7 | 177.0 | 820.3 | 701.6 |
| 2007 | 844.5 | 194.4 | 839.2 | 709.3 |
| 2008 | 841.6 | 212.3 | 856.9 | 719.9 |
| 2009 | 828.7 | 219.4 | 864.9 | 723.6 |
| 2010 | 808.4 | 228.4 | 874.4 | 729.4 |
| 2011 | 812.5 | 242.0 | 884.8 | 732.1 |
| 2012 | 807.8 | 257.5 | 886.8 | 736.6 |
| 2013 | 809.1 | 273.1 | 904.6 | 743.4 |
| 2014 | 817.1 | 283.3 | 919.8 | 751.3 |
| 2015 | 821.1 | 288.8 | 928.3 | 756.0 |
| 2016 | 831.2 | 294.0 | 935.2 | 759.9 |
| 2017 | 836.6 | 305.0 | 938.9 | 763.8 |
| 2018 | 836.3 | 309.1 |  | 767.6 |

**Table S4.** Age standardized childhood leukemia incidence rate (ASR) per 100,000 by country vs intensity of the total magnetic field of the Earth. Leukemia statistics data are from the International Agency for Research on Cancer. The magnetic field intensity calculated for each country using the [World Magnetic Model (WMM)](https://www.ngdc.noaa.gov/geomag/WMM/) should be considered for an estimation purpose only since it varies across a particular country.

| **Country** | **ASR, per 100,000** | **Intensity of Total magnetic field, nT** |
| --- | --- | --- |
| PERU. Lima (2010-2012) | 6.3 | 24,902.50 |
| ECUADOR. 5 registries (1993-2013) | 6.3 | 27,912.80 |
| CHILE. 4 registries (1998-2012) | 6.2 | 23,033.30 |
| MALTA (1994-2013) | 6.1 | 44,761.10 |
| CROATIA (2001-2014) | 6.0 | 48,130.80 |
| ITALY. 2 paediatric registries (1998-2011) | 5.9 | 47,192.70 |
| COLOMBIA. 4 registries (1992-2013) | 5.8 | 29,678.90 |
| CYPRUS. South-West (1998-2012) | 5.8 | 46,332.20 |
| PHILIPPINES. 2 registries (1993-2012) | 5.6 | 41,132.50 |
| COSTA RICA (1993-2012) | 5.6 | 33,851.50 |
| AUSTRALIA (1992-2014) | 5.6 | 54,391.20 |
| NEW ZEALAND (1993-2012) | 5.5 | 56,812.40 |
| USA. Hawaii (1993-2012) | 5.5 | 34,755.20 |
| CANADA. 9 registries (1992-2013) | 5.5 | 57,708.00 |
| GERMANY. paediatric (1996-2012) | 5.5 | 49,455.70 |
| GREECE. paediatric specialized (1996-2014) | 5.4 | 46,662.10 |
| USA. SEER 18 (1993-2012) | 5.4 | 51,637.50 |
| SWITZERLAND. paediatric (1990-2013) | 5.3 | 48,114.90 |
| SWEDEN (1990-2011) | 5.2 | 52,795.30 |
| LITHUANIA (2000-2012) | 5.1 | 51,409.30 |
| NORWAY (1990-2013) | 5.1 | 51,897.50 |
| AUSTRIA (1990-2012) | 4.9 | 48,755.10 |
| CHINA. 6 registries (1990-2013) | 4.9 | 54,576.60 |
| BELGIUM (2004-2013) | 4.9 | 49,017.20 |
| NETHERLANDS (1993-2013) | 4.9 | 49,495.30 |
| LEBANON (2008-2010) | 4.8 | 46,184.80 |
| IRELAND (1994-2012) | 4.8 | 49,368.20 |
| SPAIN. 2 paediatric registries (1991-2013) | 4.8 | 44,981.00 |
| UKRAINE (2002-2012) | 4.8 | 56,699.90 |
| CZECH REPUBLIC (1990-2012) | 4.8 | 49,424.90 |
| UK (2000-2011) | 4.6 | 49,529.90 |
| RUSSIAN FEDERATION. 2 registries (1998-2015) | 4.6 | 61,780.90 |
| FRANCE. paediatric (2000-2012) | 4.6 | 47,684.20 |
| THAILAND. 6 registries (1993-2013) | 4.5 | 43,384.70 |
| PORTUGAL (1991-2012) | 4.5 | 44,419.80 |
| JAPAN. 8 registries (1990-2013) | 4.5 | 47,497.10 |
| BELARUS. paediatric (1990-2014) | 4.4 | 51,002.3 |
| JORDAN (2000-2012) | 4.4 | 44,771.4 |
| HUNGARY. paediatric (1991-2014) | 4.4 | 48,697.1 |
| KUWAIT (1994-2012) | 4.4 | 45,225.9 |
| BAHRAIN (1998-2012) | 4.3 | 44,074.4 |
| BULGARIA (1990-2013) | 4.3 | 47,883.1 |
| SLOVENIA (1991-2012) | 4.3 | 48,040.1 |
| ICELAND (1990-2014) | 4.3 | 52,308.8 |
| ESTONIA (1990-2012) | 4.2 | 52,111.5 |
| POLAND (2001-2013) | 4.2 | 49,949.9 |
| SLOVAKIA (1990-2009) | 4.1 | 49,093.7 |
| ISRAEL (1990-2012) | 3.8 | 44,931.4 |
| FRANCE. 3 registries (1990-2012) | 3.6 | 47,422.7 |

# 3. Supplementary data analysis

**3.1. Incidence rate**

**Table S5.** Descriptive statistics of the childhood leukemia incidence rate variability for the period 1990-2018.

|  | *USA* | *Russia* | *Canada* | *Australia* |
| --- | --- | --- | --- | --- |
| Mean | 4.18 | 3.55 | 5.03 | 5.27 |
| Stand. Error | 0.07 | 0.11 | 0.10 | 0.07 |
| Median | 4.13 | 3.54 | 5.12 | 5.24 |
| Stand. Deviation | 0.36 | 0.59 | 0.48 | 0.38 |
| Sample Variance | 0.13 | 0.35 | 0.23 | 0.15 |
| Kurtosis | -0.16 | -0.91 | -0.63 | -0.88 |
| Skewness | 0.20 | 0.26 | 0.08 | 0.21 |
| Range | 1.50 | 2.22 | 1.75 | 1.40 |
| Minimum | 3.41 | 2.43 | 4.11 | 4.59 |
| Maximum | 4.91 | 4.65 | 5.87 | 5.99 |
| Largest | 4.91 | 4.65 | 5.87 | 5.99 |
| Smallest | 3.41 | 2.43 | 4.11 | 4.59 |

Details of the descriptive statistics of the incidence rates discussed in the manuscript are provided in **Table S5**.

The manuscript shows the linear regression to illustrate a rising trend in the childhood leukemia (see Figure 2 of the manuscript for the approximation formulas). In **Figures S1-S4**, details of the linear approximation are shown for the USA, Russia, Canada, and Australia, respectively. The approximation line in the upper panel is red, the confidence interval is blue, and the bottom panel shows residual values (difference between the model and data). R-Squared (R² or the coefficient of determination) is shown in each figure. It assesses the goodness of fit of a regression model, measures the strength of the relationship between your model and the dependent variable on a convenient 0 – 100% scale. The regression statistics, the estimated slope along with its standard error, and lower/upper 95% confidence intervals (CIs) are given as tables in **Figures S1-S4**.

One may be interested in fitting the disease rate curves to a higher-degree polynomial, for which polynomial regression constructing a polynomial equation to approximate the relationship between variables in a dataset can be employed. The method follows the principle: *y*=A_0_+A_1_*x*^1^+A_2_*x*^2^+…+A_n_*x*^n^, where *y* represents the dependent variable, *x* is the independent variable, a_0…_a_n_ are the coefficients, and *n* denotes the polynomial order. The Levenberg-Marquardt algorithm (S5) is utilized below for curve-fitting, employed to accurately fit a nonlinear model to observed data. This iterative algorithm adjusts model parameters to minimize the difference between observed data and model predictions, employing a combination of gradient descent and Newton's methods. (see [https://en.wikipedia.org/wiki/Levenberg%E2%80%93Marquardt_algorithm](https://en.wikipedia.org/wiki/Levenberg–Marquardt_algorithm) for details).

**Figures** **S5-S8** show results of the fitting of the same incidence rate data, displaying the polynomial fit (red) and the 95% confidence band (blue) in the upper panels, and the residuals– in the bottom panels, analogous to **Figures S1-S4**.


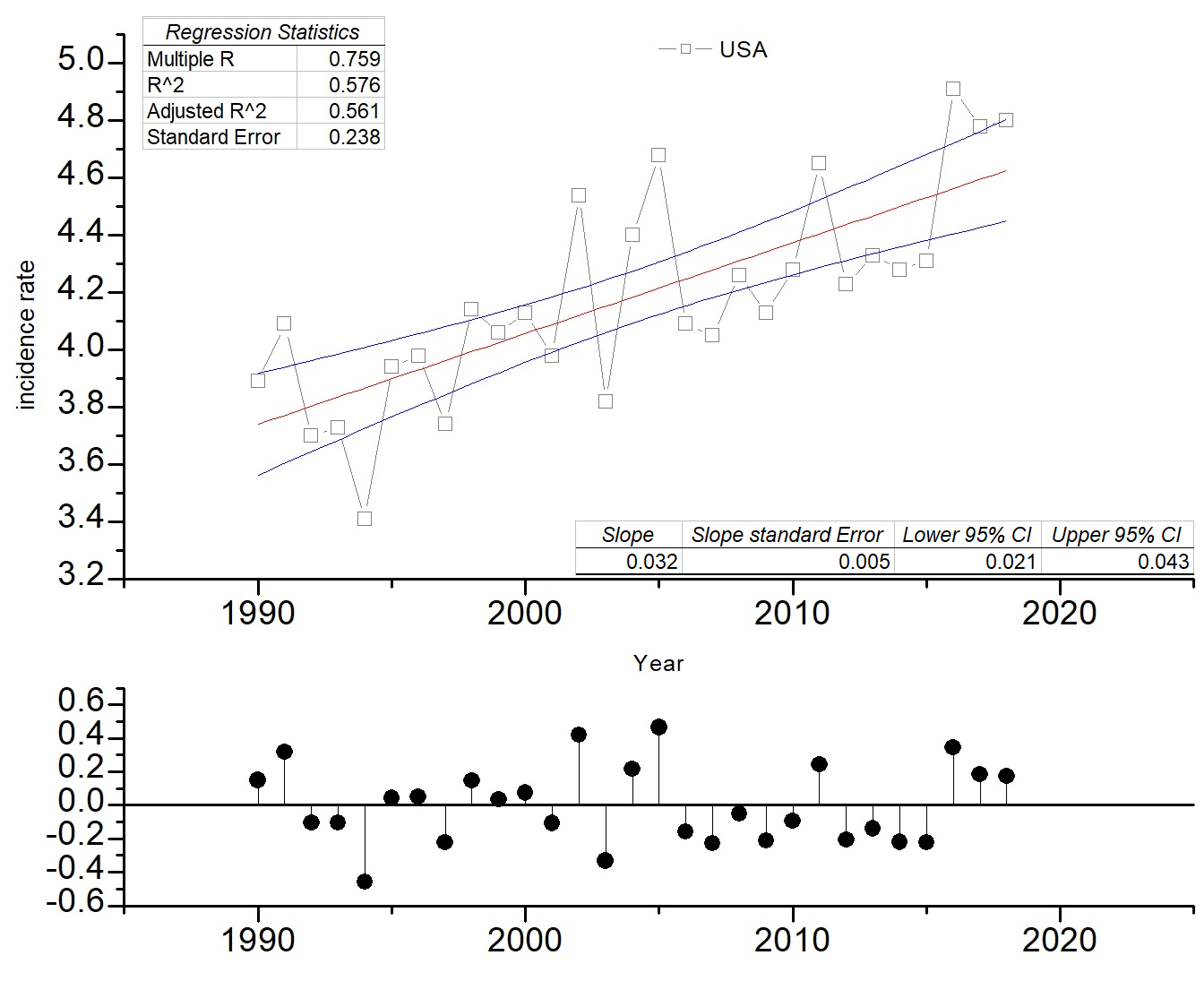


**Figure S1.** Linear regression analysis of the annual leukemia incidence rate data in the USA. Upper panel displays squares representing the incidence rate, overlaid with a red linear regression line and a blue confidence band. Bottom panel depicts black dots representing residual values. Key regression statistics parameters are presented in the upper panel and summarized in Table S5.


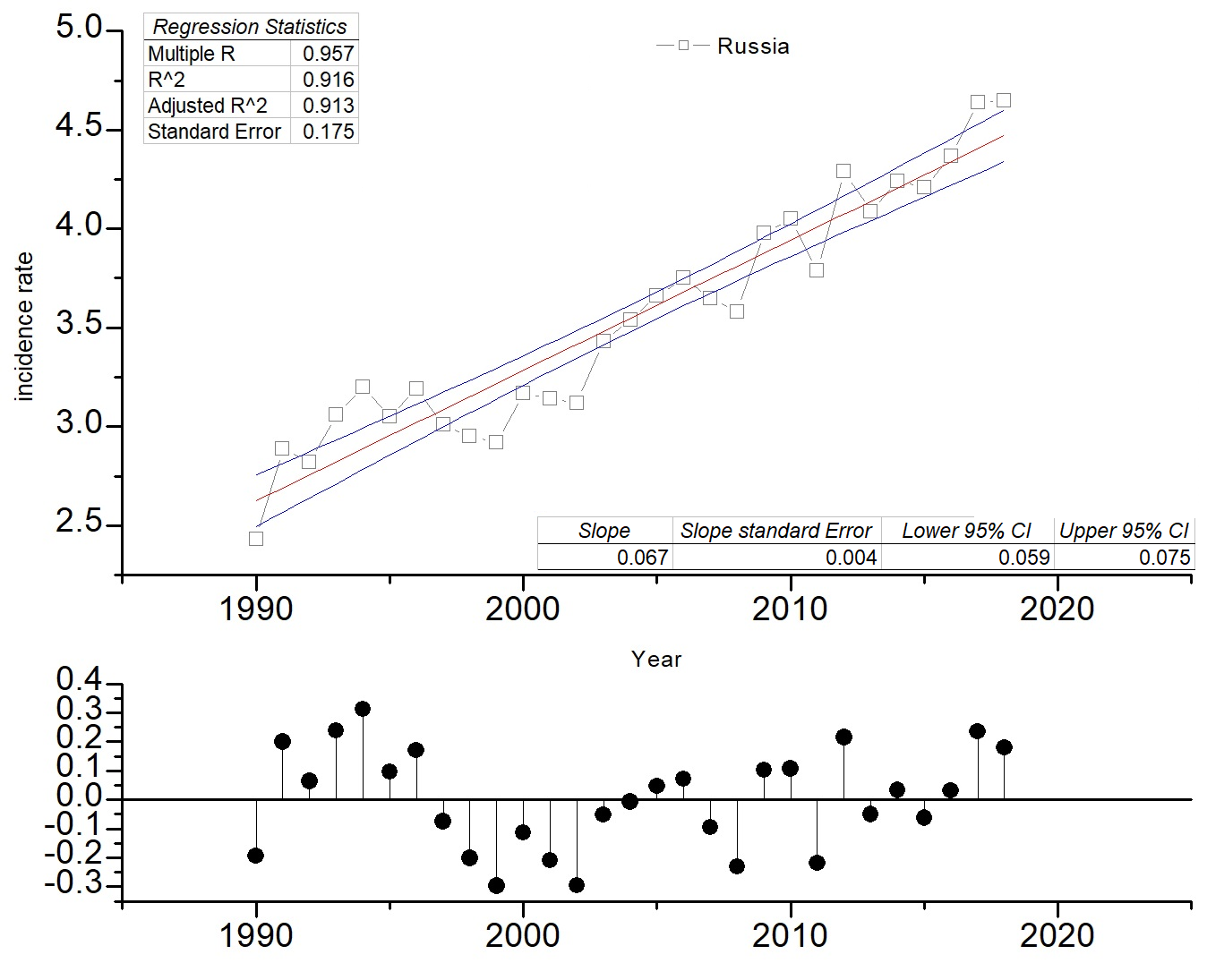


**Figure S2.** Linear regression analysis of the leukemia incidence rate in Russia. Upper panel: squares – incidence rate, red- linear regression line, blue – confidence band, black dots in the bottom panel are residual values. Key regression statistics parameters are presented in the upper panel and summarized in Table S5.


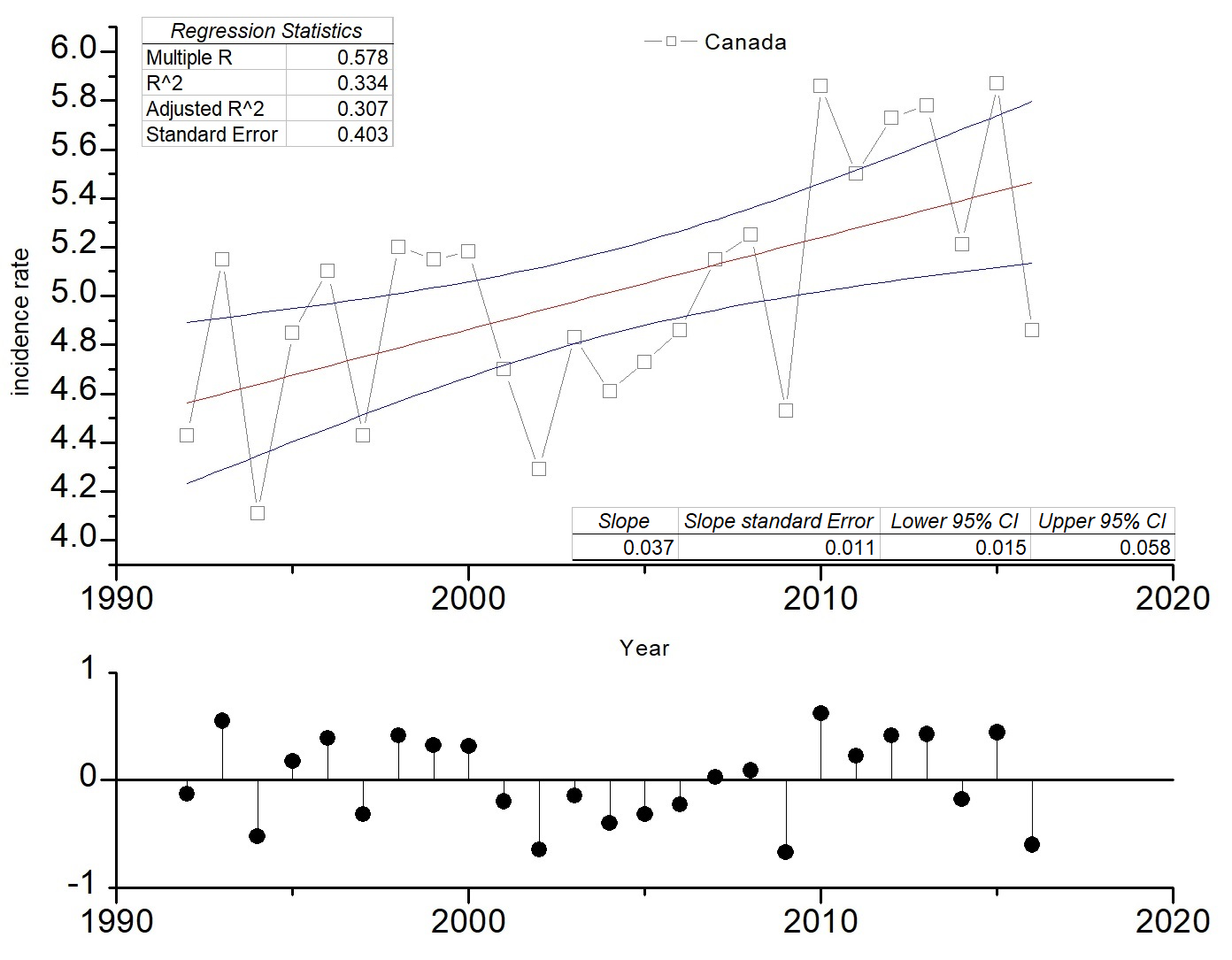


**Figure S3.** Linear regression analysis of the leukemia incidence rate in Canada. Upper panel: squares – incidence rate, red- linear regression line, blue – confidence band, black dots in the bottom panel are residual values. Key regression statistics parameters are presented in the upper panel and summarized in Table S5.


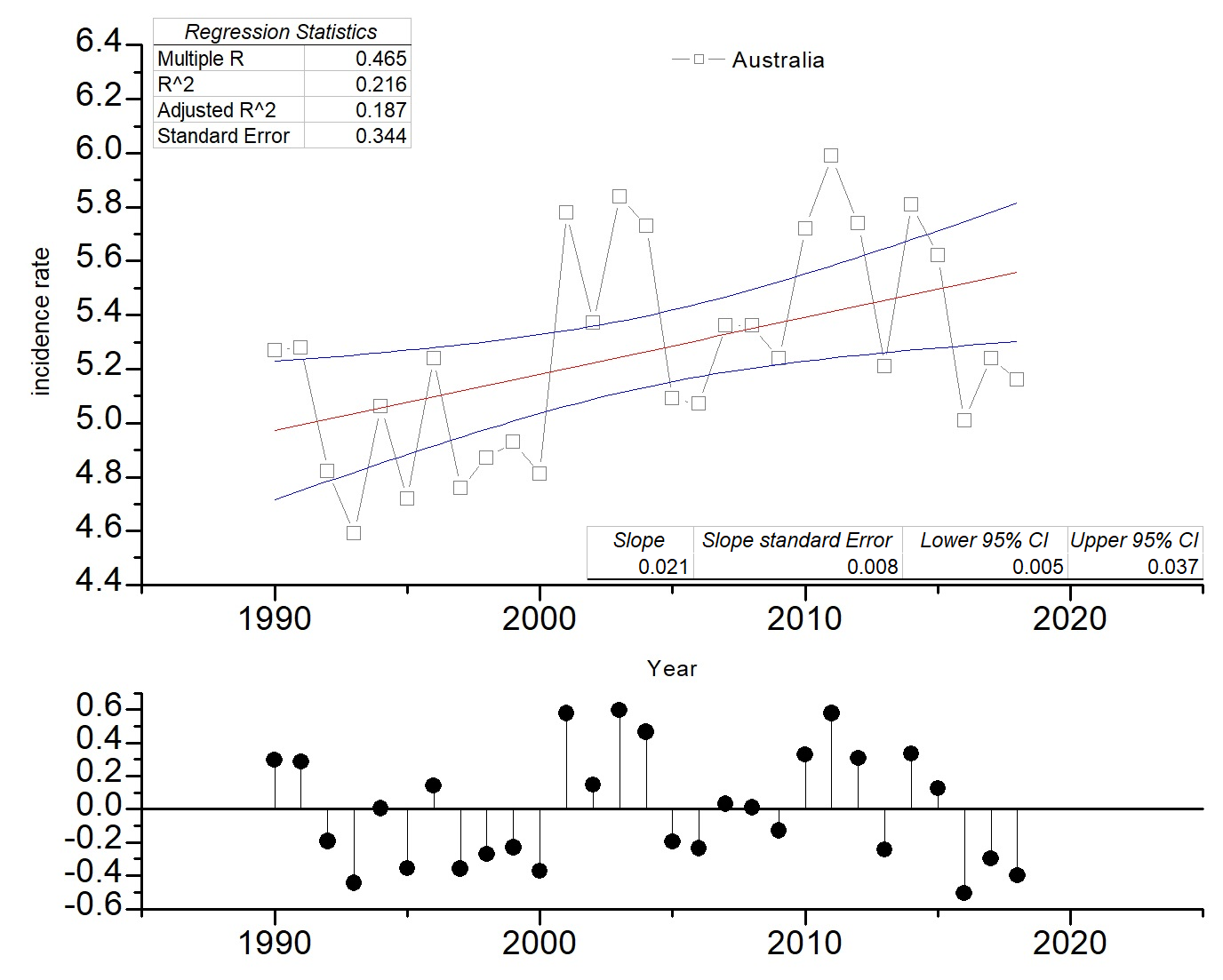


**Figure S4.** Linear regression analysis of the leukemia incidence rate in Australia. Upper panel: squares – incidence rate, red- linear regression line, blue – confidence band, black dots in the bottom panel are residual values. Key regression statistics parameters are presented in the upper panel and summarized in Table S5.


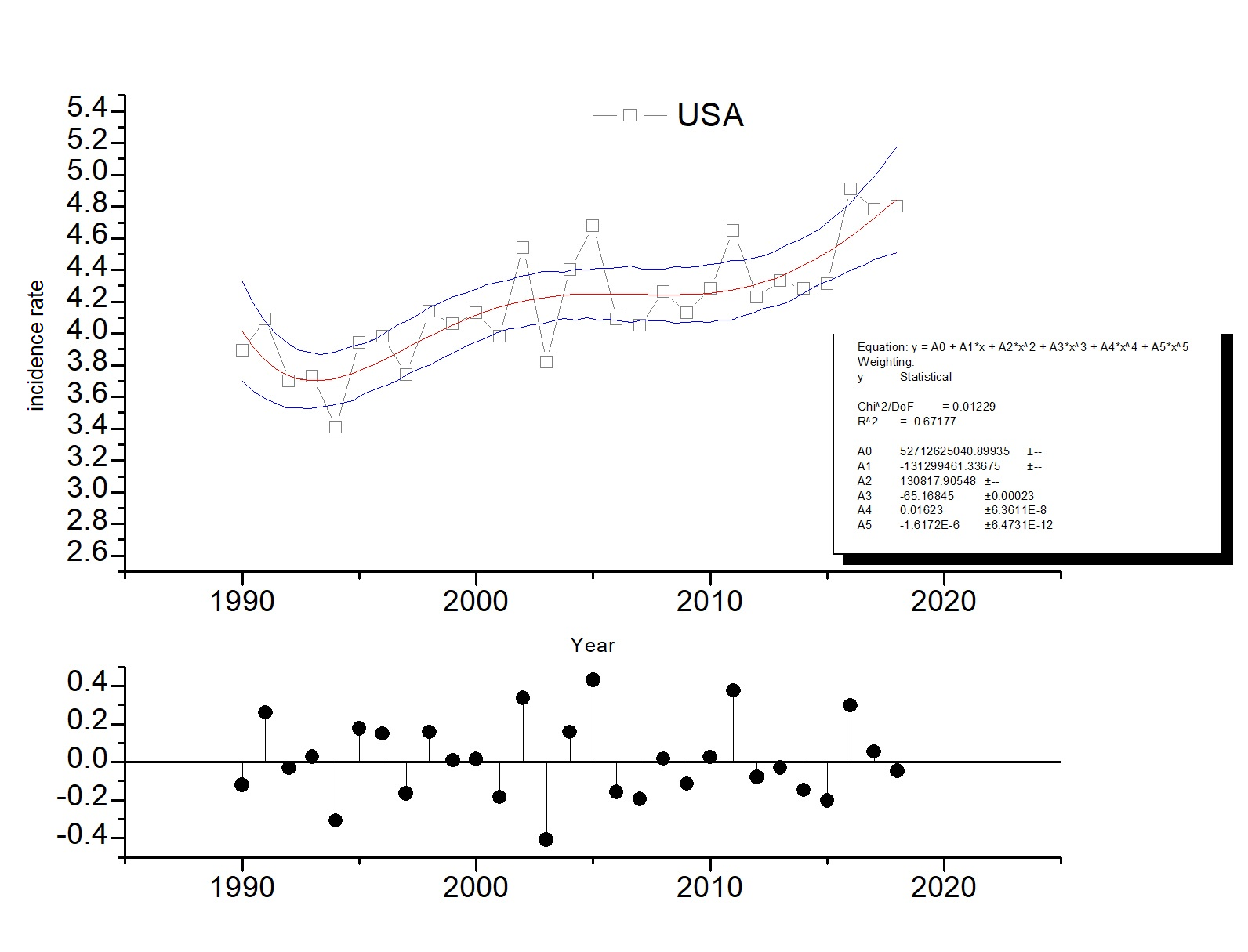


**Figure S5.** Approximation of the childhood leukemia incidence rate in the USA by the fifth-degree polynomial. Squares – incidence rate, red – polynomial, blue – confidence interval, black dots in the bottom panel are residuals. Coefficients of the equation *y*=A_0_+A_1_*x*^1^+A_2_*x*^2^+A_n_*x*^3^+A_1_*x*^4^+A_2_*x*^5^ are given in the table in the upper panel, where *y* is the incidence rate, and *x* is the year. The key regression statistics parameters are shown in the upper panel.


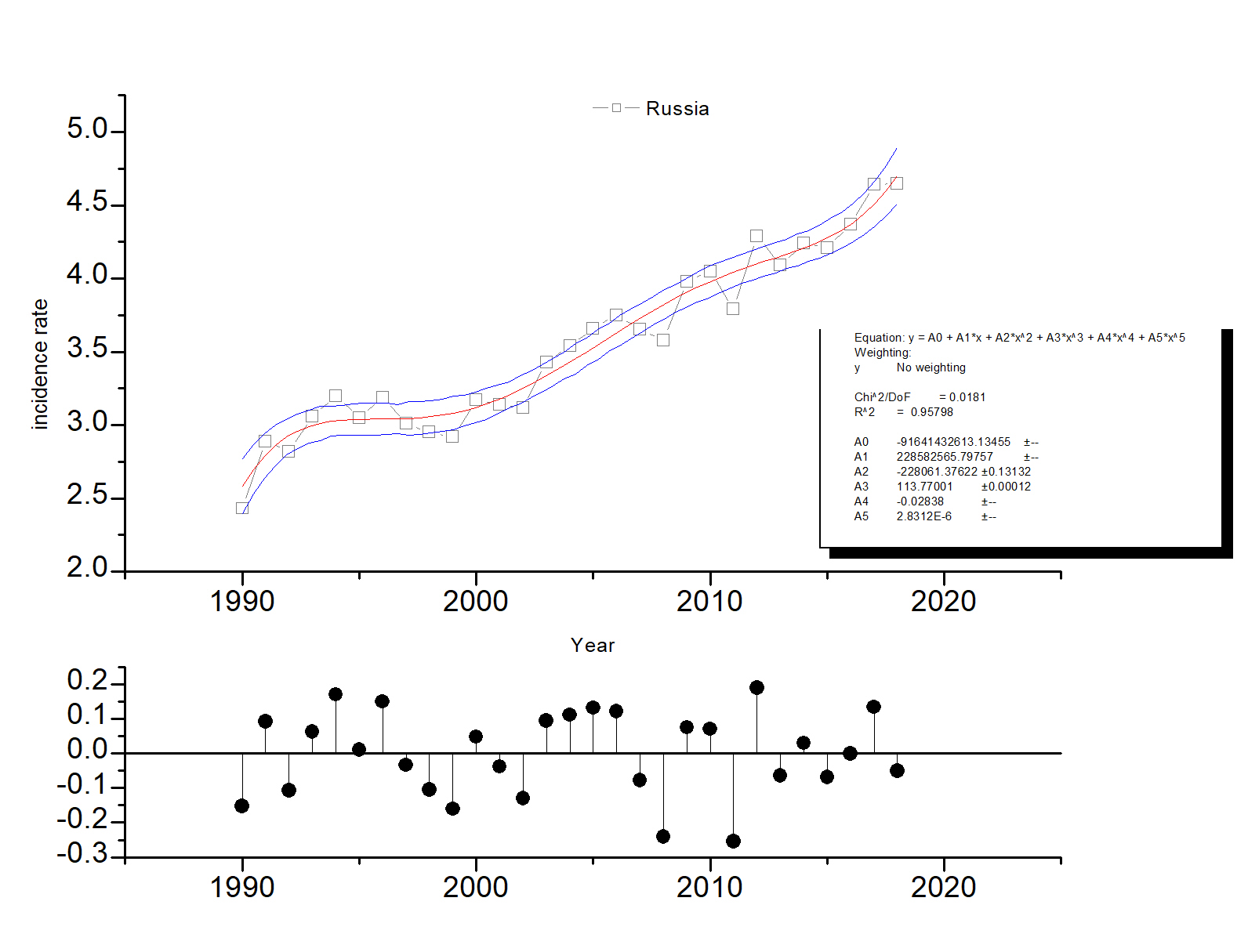


**Figure S6.** Approximation of the childhood leukemia incidence rate in Russia by the fifth-degree polynomial. Squares – incidence rate, red – polynomial, blue – confidence interval, black dots in the bottom panel are residuals. Coefficients of the equation *y*=A_0_+A_1_*x*^1^+A_2_*x*^2^+A_n_*x*^3^+A_1_*x*^4^+A_2_*x*^5^ are given in the table in the upper panel, where *y* is the incidence rate, and *x* is the year. Key regression statistics parameters are shown in the upper panel.


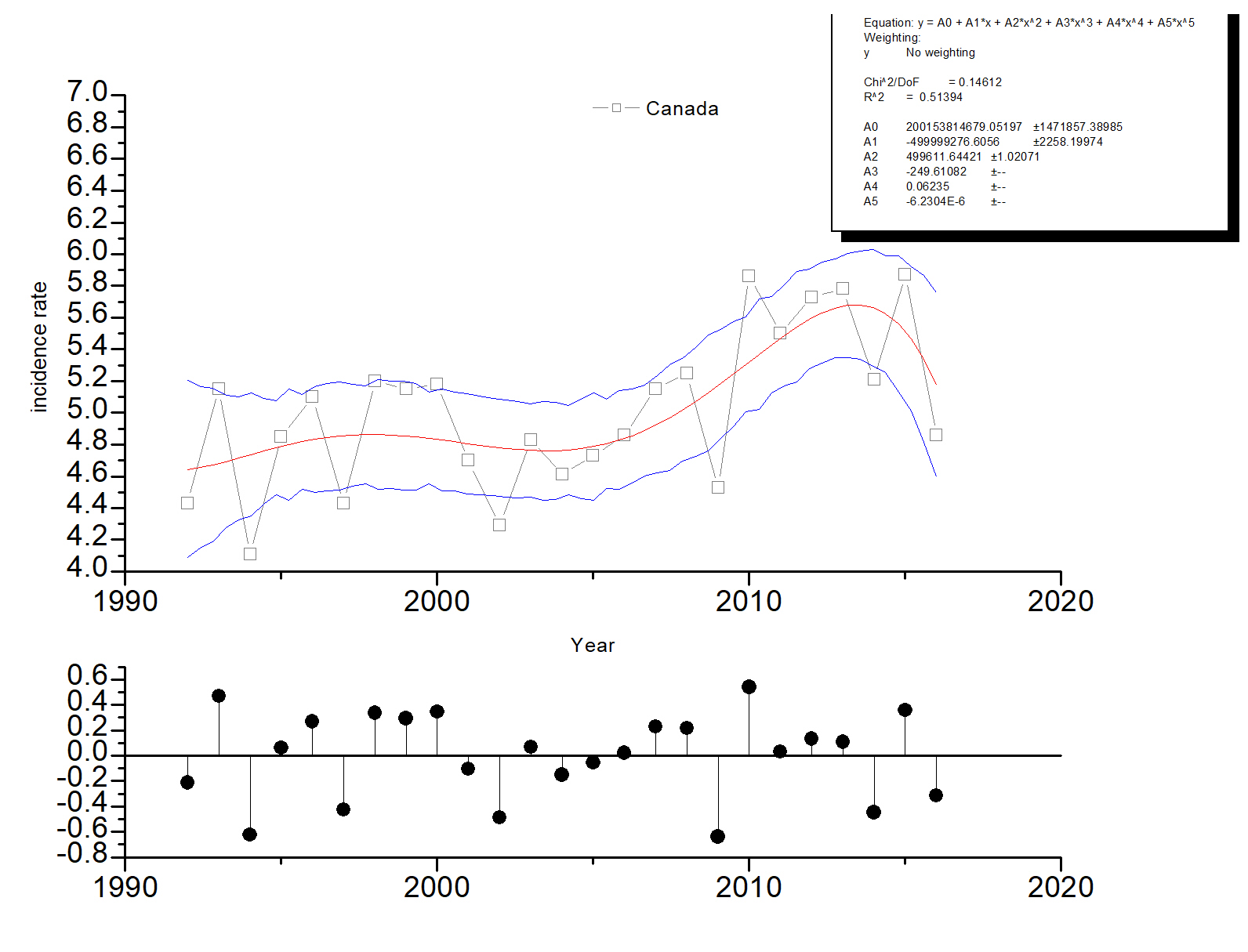


**Figure S7.** Approximation of the childhood leukemia incidence rate in Canada by the fifth-degree polynomial. Squares – incidence rate, red – polynomial, blue – confidence interval, black dots in the bottom panel are residuals. Coefficients of the equation *y*=A_0_+A_1_*x*^1^+A_2_*x*^2^+A_n_*x*^3^+A_1_*x*^4^+A_2_*x*^5^ are given in the table in the upper panel, where *y* is the incidence rate, and *x* is the year. Key regression statistics parameters are shown in the upper panel.


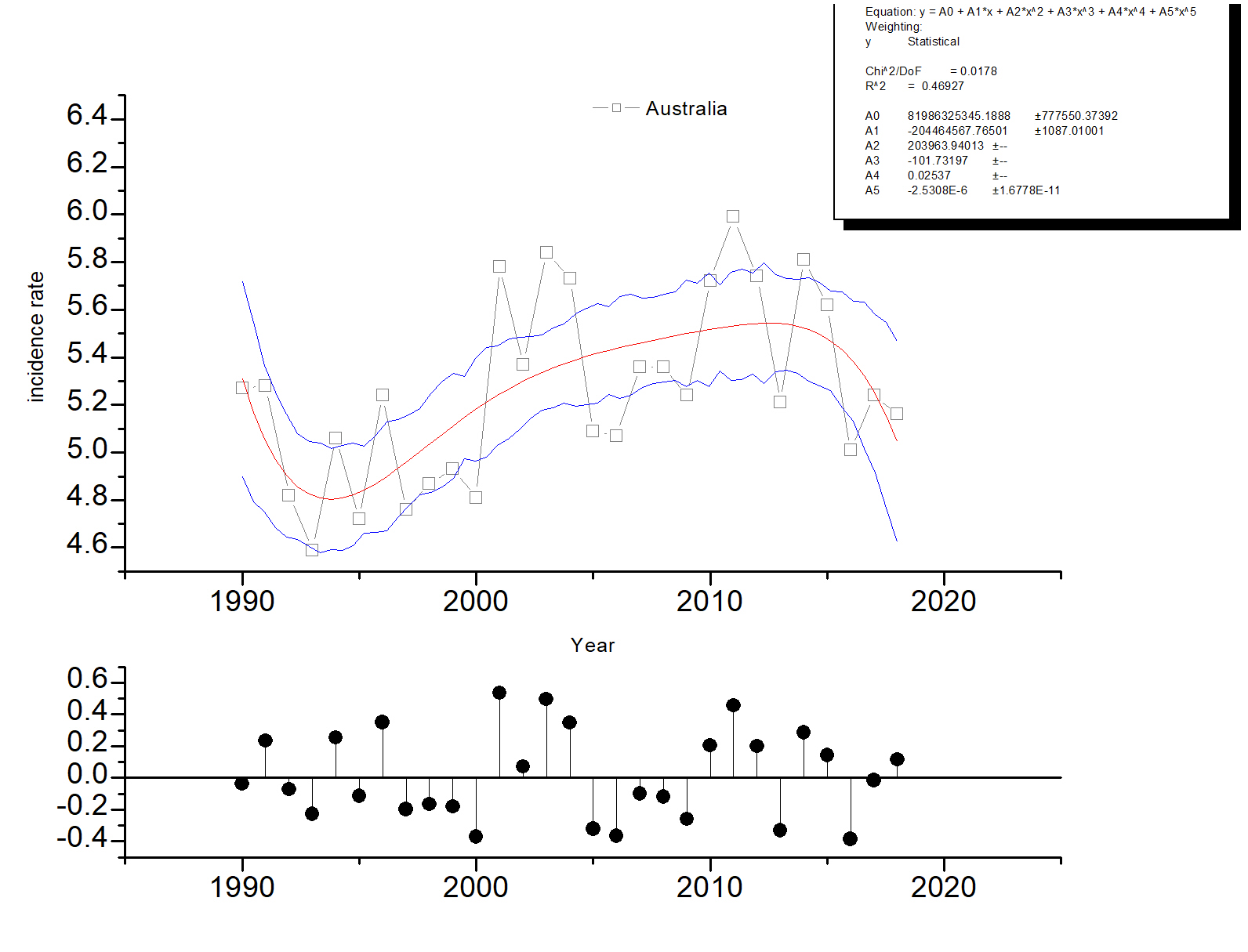


**Figure S8.** Approximation of the childhood leukemia incidence rate in Australia by the fifth-degree polynomial. Squares – incidence rate, red – polynomial, blue – confidence interval, black dots in the bottom panel are residuals. Coefficients of the equation *y*=A_0_+A_1_*x*^1^+A_2_*x*^2^+A_n_*x*^3^+A_1_*x*^4^+A_2_*x*^5^ are given in the table in the upper panel, where *y* is the incidence rate, and *x* is the year. Key regression statistics parameters are shown in the upper panel.

**3.2. Possible tie with atmospheric ozone, cosmic rays and magnetic field**

**Figure S9** illustrates a possible impact of cosmic rays on initiation of leukemia in children via ozone destruction in the lower atmosphere. The ozone concentration in New Jersey anti-correlates with the CR flux. The ozone concentration curve in **Figure S9** is shifted for one year with respect to the leukemia incidence curve and shown in a reverse order. The correlation coefficient between the two rows upon a shift of the ozone concentration curve for one year ahead is -0.6. Data for ozone are provided by New Jersey Department of Environmental Protection Bureau of Air Monitoring in its 2020 report: <https://www.nj.gov/dep/airmon/pdf/2020-nj-aq-report.pdf> .

**Figure S10** depicts a regression analysis based on the data from Figure 8 in the manuscript, which examines leukemia rate statistics across 49 countries in relation to the Earth's total magnetic field intensity, as detailed in Table S4 above. Unlike Figure 8, **Figure S10** does not categorize the data into two groups. While Figure 8 specifically emphasizes countries most affected by cosmic rays due to insufficient magnetic field shielding, as explained in section 3.3.1 of the manuscript, in **Figure S10**, the ASR vs F curve is approximated using a 5th-order polynomial, yielding a satisfactory R^2^ level of 0.6. As mentioned in the manuscript, the plateau observed at the intermediate level of F indicates countries with optimal protection against cosmic rays provided by the magnetosphere.


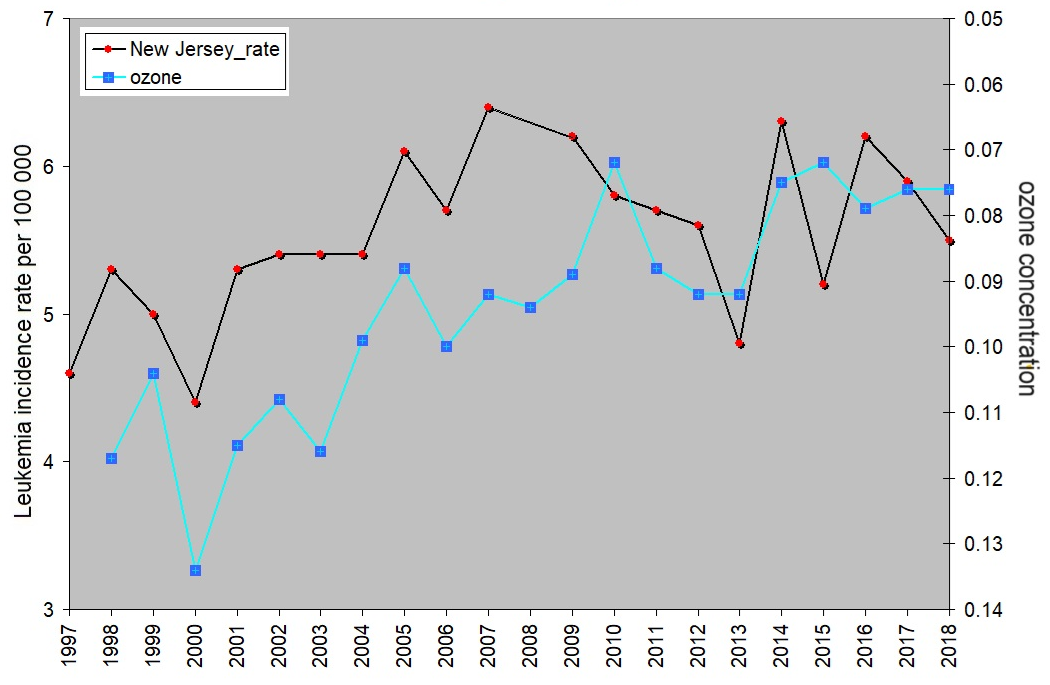


**Figure S9.** Daily maximum 8-hour-average ozone concentration (parts per million - ppm) vs childhood leukemia incidence rate per 100,000 in New Jersey, US.


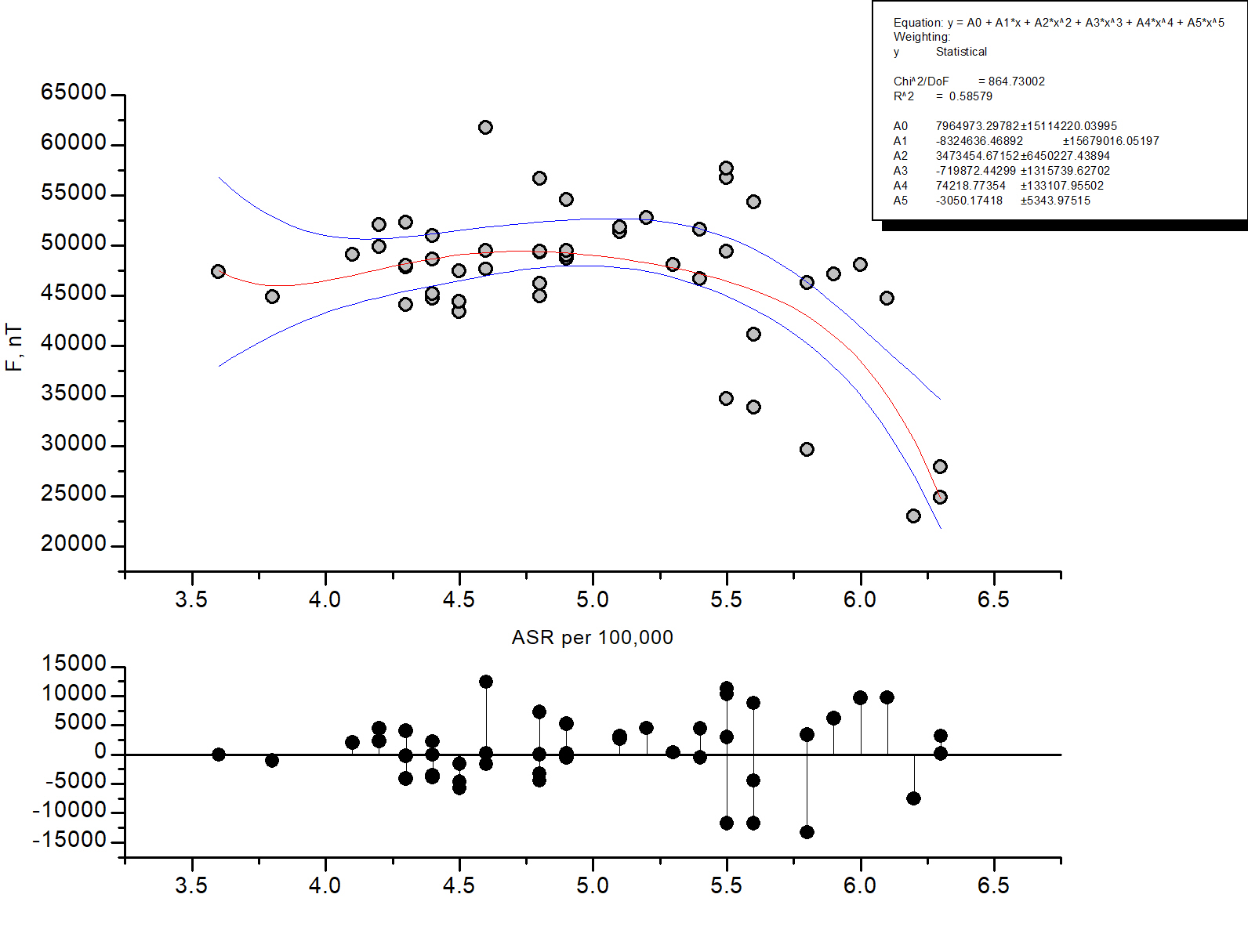


**Figure S10.** ASR statistics for 49 countries vs intensity of the total magnetic field of the Earth F (see Fig. 8 of the manuscript) but showing the approximation with 5th-order polynomial.

REFERENCES

S1. Hunger SP, Mullighan CG (2015) Acute lymphoblastic leukemia in children. New England *Journal of Medicine. Massachussetts Medical Society*. 373(16):1541–1552. https://doi.org/10.1056/nejmra1400972

S2. Cooper SL, Brown PA (2015) Treatment of pediatric acute lymphoblastic leukemia. *Pediatr Clin North Am* 62(1):61-73. doi: 10.1016/j.pcl.2014.09.006

S3. Swerdlow SH, Campo E, Pileri SA, Harris NL, Stein H, Siebert R, Advani R, Ghielmini M, Salles GA, Zelenetz AD, Jaffe ES (2016 ) The 2016 revision of the World Health Organization classification of lymphoid neoplasms. *Blood* 127(20):2375-90. doi: 10.1182/blood-2016-01-643569

S4. Creutzig U, van den Heuvel-Eibrink MM, Gibson B, Dworzak MN, Adachi S, de Bont E, Harbott J, Hasle H, Johnston D, Kinoshita A, Lehrnbecher T, Leverger G, Mejstrikova E, Meshinchi S, Pession A, Raimondi SC, Sung L, Stary J, Zwaan CM, Kaspers GJ, Reinhardt D; AML Committee of the International BFM Study Group (2012) Diagnosis and management of acute myeloid leukemia in children and adolescents: recommendations from an international expert panel. *Blood* 120(16):3187-205. doi: 10.1182/blood-2012-03-362608

S5. Marquardt DW (1963) An Algorithm for Least-Squares Estimation of Nonlinear Parameters *Journal of the Society for Industrial and Applied Mathematics* 11(2):431-441. https://www.jstor.org/stable/2098941

**
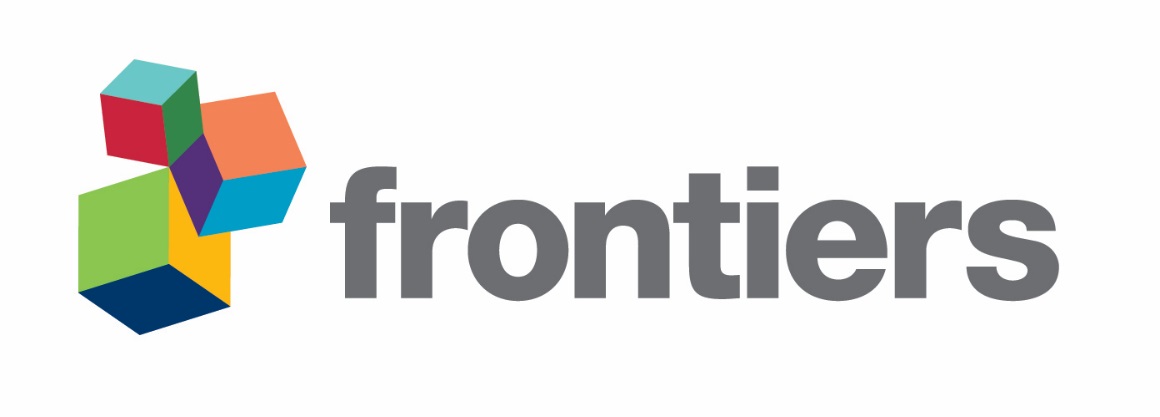
**
